# Machine learning algorithm for estimating and optimizing the phytochemical content and physicochemical properties of okra slices in an infrared heating system

**DOI:** 10.1016/j.fochx.2025.102248

**Published:** 2025-01-30

**Authors:** Hany S. El-Mesery, Ahmed H. ElMesiry, Evans K. Quaye, Zicheng Hu, Ali Salem

**Affiliations:** a School of Energy and Power Engineering, Jiangsu University, Zhenjiang 212013, China; b Agricultural Engineering Research Institute, Agricultural Research Center, Dokki 12611, Giza, Egypt; c Faculty of Computer Science and Engineering, New Mansoura University, 35742, Egypt; d Civil Engineering Department, Faculty of Engineering, Minia University, Minia 61111, Egypt; e Structural Diagnostics and Analysis Research Group, Faculty of Engineering and Information Technology, University of Pécs, Pécs 7622, Hungary

**Keywords:** Infrared drying of Okra, Optimization, Machine Learning in Food Processing, Self-Organizing Map, physicochemical properties

## Abstract

This study investigates how different air temperatures and infrared intensities affect the physicochemical properties of dried okra at different airflow rates. The model was developed using machine learning, and Okra's physicochemical properties were optimized using a self-organizing map (SOM). The results showed that higher infrared intensity and air temperature improved rehydration and colour but reduced water activity and vitamin C levels. In contrast, faster airflow helped preserve quality by counteracting the negative effects of higher temperatures and infrared intensity. The SOM algorithm identified five optimal drying conditions, revealing that lower temperatures, lower infrared intensity, and higher airflow provided optimal conditions for improving the quality of okra slices. Interestingly, the machine learning model's predictions closely matched the test data sets, providing valuable insights for understanding and controlling the factors affecting okra drying performances. This study used machine learning to optimize the drying process of okra, a new approach for improving food drying techniques. It offers valuable insights for the food industry in its quest to improve efficiency without sacrificing product quality.

## Highlights

• Okra is a highly perishable vegetable crop.
• ML used to predict the optimal process conditions in drying okra slices.
• Infrared intensity, airflow, and temperature influenced okra's physicochemical properties.
• A hybrid system is a more suitable drying technique to obtain high-quality okra.
• This research highlights the ML in refining food-drying processes.

## 1 Introduction

Vegetables and fruits are vital components of a balanced diet. Several clinical and epidemiological studies have linked diets rich in fruits and vegetables to reduced risks of coronary heart, metabolic and degenerative diseases, cancers, and cardiovascular diseases. Their strong antioxidant activity is attributed mainly to their fibre content, minerals, vitamins, and phytochemicals, such as sterols, chlorophylls, carotenoids, polyphenols, anthocyanins, and flavonoids. (Samakradhamrongthai et al., 2022). Okra (*Abelmoschus esculentus L.*) is also known as "Bhindi" in India and the United States and "gumbo" in Africa. It can be eaten fresh, cooked, or added to soups, stews, and salads. Additionally, the unripe fruit is utilized as a diuretic and to cure oral conditions in traditional medicine. It is abundant in fibre, vitamin B6, protein, minerals, vitamin C, antioxidants, flavonoids, folic acid, and polysaccharides.

Fresh fruits and vegetables are prone to rapid spoilage due to their moisture content, typically about 80 %, and without proper handling, they could deteriorate in a short period (EL-Mesery et al., 2022). Food items are dried to reduce packaging needs, transit weights, and storage periods while preventing deterioration and microbial development. Most fresh okras are stored because they are naturally perishable due to their intense respiratory activity and high moisture content. Dried fruits and vegetables are common sources of raw materials for sweet and baking industries, confectionary, and distilling, and for children and infants, they serve as food. They are also used in the beverage industry as fruit and vegetable powders, serving as intermediate ingredients. Additionally, they function as food additives that enhance nutritional content and additives for flavours or colorants in yogurts, fruit bars, and ice creams (Sekar et al., 2023).

Drying is an old and diverse food processing method. It is a complex process involving heat and mass transfer and demands precise control. To dry a moist substance implies that water in the material, both free and loosely bound, evaporates into the surrounding air. As an energy-intensive operation, drying accounts for 10-25% of the total energy utilized in the food processing process globally (Y. Huang et al., 2025). To increase shelf life, improve storage stability, reduce weight during transportation, and minimize packing needs, fruits and vegetables are often dried. Fruits and vegetables have been dried using various processing methods. Although dehydrated items made by hot air drying have a longer shelf life of up to a year, their quality is much worse than that of the original meal. One method of moderate dehydration that is perfect for creating high-value goods is infrared drying (Akhoundzadeh Yamchi et al., 2024).

Comparing the infrared heating process to conventional drying techniques under comparable drying conditions reveals several benefits, such as uniform temperature, better food quality, drying time, and high energy cost efficiency. Only the food receives the heat released by infrared radiation; the air around it remains unaltered. Emissions impact the detected sample by penetration, converted into plausible heat (Gu et al., 2022). Hybrid drying techniques utilize more than one drying technique or several modes of heat transfer to obtain the required functionality of dryness without compromising product quality. Numerous studies have examined several hybrid drying technologies that combine several drying systems. These techniques are often less costly than fully complete drying technologies in the large-scale food industry (El-Mesery, Hu, et al., 2024; El-Mesery, Qenawy, et al., 2024).

Colour, texture, sensory qualities, and the loss of water-soluble vitamins (mostly ascorbic acid) are just a few of food's physical and chemical characteristics that may be impacted by hybrid drying. They are also crucial markers when assessing dried fruits and vegetables. Colour determines food's appearance, processing, and consumer appeal. When a food product is presented for sale, its visual appearance is the first impression it makes on the consumer. During thermal processing, colour changes occur due to chemical reactions within the food. These reactions can involve the degradation of pigments, particularly carotenoids and chlorophyll, browning processes such as the Maillard reaction between hexoses and amino acids, and the oxidation of ascorbic acid (Boateng et al., 2021). Small colour changes, keeping close to that of the fresh product, are best as this is often seen as an indicator of high-quality dry goods. Enzymatic and non-enzymatic alterations of pectin, a key cell wall polysaccharide, primarily cause textural degradation of thermally processed biomaterials. In addition, measuring the firmness of foods during processing is important as it provides valuable information about changes in texture (Jiang et al., 2024).

The application of machine learning in food processing has been heavily studied over the past decade, especially in modeling and drying process optimization. The artificial neural networks (ANN) model is a machine learning-based strategy that uses information to forecast complex system outcomes like drying technologies. Artificial neural networks, or ANNs, are now crucial for examining different interactions in various domains, especially food processing. When compared to other traditional modeling techniques, ANN is very popular because it is simple to use, has excellent forecasting capabilities, allows for the reduction or increase of input and output variables, and can be used to predict more than two output variables (El-Mesery, Hu, et al., 2024; El-Mesery, Qenawy, et al., 2024). Machine learning in food processing has been heavily applied over the past decade, especially in modelling and drying process optimization. Various machine-learning models, including ANN, SVM, and RF, have been utilized to predict drying times, minimize energy consumption, and assess final product quality. Machine learning has also been explored for real-time monitoring and control of food processing to enhance efficiency and quality. While these recent advances have been in machine learning, many studies rely on a very limited dataset and simple models, which cannot represent the fundamental interactions occurring in drying. Moreover, most machine learning (ML) models are not integrated into real-time systems designed for adaptive control; thus, achieving simultaneous optimization of multiple variables remains challenging (Miraei Ashtiani & Martynenko, 2024). There remains a significant gap in incorporating ML models, particularly concerning infrared (IR) drying processes, for real-time monitoring and multi-variable optimization. Although some studies use machine learning approaches to predict drying time or improve the energy efficiency of traditional drying methods, such as convective drying and microwave heating, minimal research is dedicated to incorporating machine learning into infrared drying. Applications of real-time monitoring and adaptive control, enabled by machine learning, during infrared (IR) drying processes have been limited. Real-time adjustments based on ML algorithms could substantially benefit energy efficiency and product quality. Therefore, machine learning can better capture these complex interactions and give more precise predictions concerning drying kinetics and product quality than the traditional approach (Dutta et al., 2025). The current study is one of the few studies that have implemented machine learning in an infrared drying system to simultaneously optimize more than one aspect of the drying process, including energy efficiency, drying time, and product quality. Unlike previous studies focused on offline optimization, the real-time control system developed in this work adjusts infrared drying parameters based on sensor data and predictions made by machine learning algorithms. This new approach addresses the shortcomings of real-time process control, which may lead to improved product quality and energy efficiency. The study increases knowledge in this area by using machine learning algorithms to understand the complex, non-linear correlations between multiple variables involved in the infrared drying process, such as food moisture content, temperature, and drying time. Although there are many studies on drying various agricultural products, no specific research is focused on drying okra slices using continuous infrared-assisted convective hot air drying and optimizing the phytochemical composition and physicochemical attributes using machine learning and ANN techniques. This study employs a continuous dryer to investigate the impact of varying infrared intensities, airflow, as well as air temperature on the phytochemical composition and physicochemical characteristics of okra slices, including (water activity, Vitamin C content, Total phenolic content (TPC), Total flavonoid content (TFC), Chlorophyll, rehydration ratio, shrinkage ratio, total colour change, and texture) during the drying process. The research also aims to develop a machine learning-based model to forecast the changes in the physicochemical quality of dried okra slices. A novel approach for discovering how to optimize the conditions of the study is offered by self-organizing maps (SOM) and Principal component analysis (PCA). These approaches are used to understand drying conditions and the relationship between okra's physicochemical properties and phytochemical content and is expected to enhance efficiency and quality in food processing.

## 2 Materials and methods

**Figure 1:**
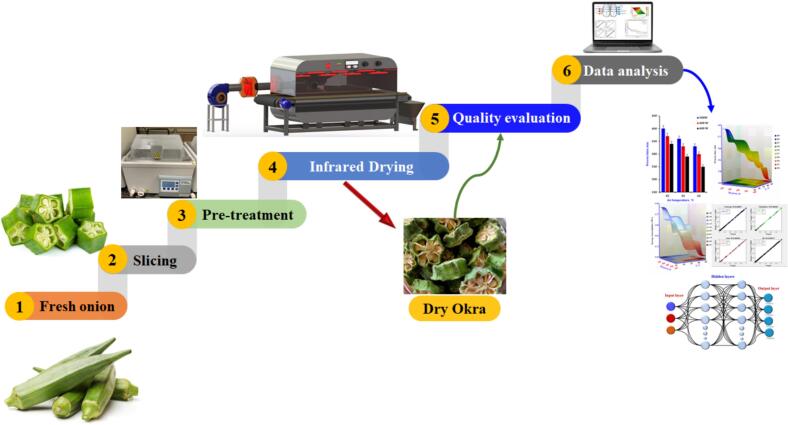
Flowchart for drying processing of okra slices.

Figure 1. depicts a complete experimental flowchart for drying and analyzing okra. This work describes the drying of okra slices using an integrated infrared and convective heating system and their effect on okra's physicochemical properties and phytochemical content.

### 2.1 Materials

Fresh okra pods (*Abelmoschus esculentus L.*) of comparable sizes, forms, and colors were bought at a local market and kept in a fridge for a day at 4 °C. Before the experiment started, the okra pods were removed and left at ambient temperature for one hour. After the inedible portions of the okra were removed, slices were cut to a thickness of about 1 cm. After blanching the okra slices at 95 °C in hot water for five minutes, the blanched pods were immediately cooled with cold water. The main aim of this treatment was to inactivate the enzymatic reactions.

### 2.2 Infrared conveyor-belt dryer

**Figure 2:**
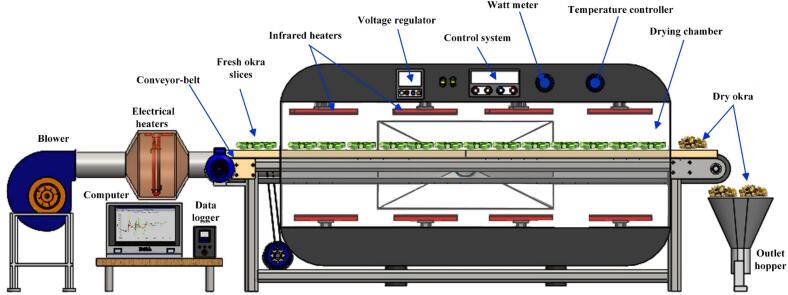
A schematic diagram of the experimental setup displays the infrared-convective conveyor belt dryer.

A schematic diagram for an infrared conveyor-belt dryer is depicted in Figure 2. The dryer, measuring 3 × 1.5 × 8 m in length, height, and weight, includes a conveyor belt system, feeder, hot air convection system, drying chamber, product collection area, and infrared heating system. The dryer was designed with a drying chamber divided into two equal sections, each measuring 0.8 × 0.8 m, constructed from 2 mm thick stainless-steel sheets. The exterior walls were shielded with an asbestos layer. A conveyor belt made of stainless steel connected the drying chambers and was employed to move samples in and out. The hot air system has a fan and two electric heaters to generate the required drying air velocity (0.2 to 4 m/s). A control unit manages these heaters to ensure a steady temperature throughout the drying process. The infrared heating system was equipped with 1000 W halogen lamps (Philips, tube type), each measuring 35.5 cm long and 0.6 cm in diameter. With a 1500-6000 W/m^2^ heating intensity range, the IR lamps were mounted on the drying chamber's upper interior surface to enable even heating. The IR temperature sources parallel the conveyor belt at a 15 cm spacing between them. A switch could halt the conveyor when the product was placed directly beneath the infrared heaters. By turning the heating systems on and off, this dryer enables the use of hot air and infrared heating separately or simultaneously. In the drying process and with an accuracy of ±1 °C, the air temperature was monitored with a T-type thermocouple.

### 2.3 Drying technique

Okra slices (300 g) were placed on a belt. The dryer was operated at airflows (AF) of 0.3, 0.5, and 1.0 m/s, drying air temperatures (T) of 35°C, 45°C, and 55°C, and infrared intensities (IR) of 0.10, 0.20, and 0.40 W/cm^2^. The experimental conditions for drying okra slices using infrared heating technology are detailed in Table 1. The dryer was run for 45 minutes until steady-state drying conditions were achieved before each drying test. The slices were dried until their moisture content dropped below 10 % (wb). The dry slices were vacuum-sealed in bags to avoid water absorption and kept at 4 °C for subsequent analysis.

**Table 1:** Experimental conditions for drying okra slices using the infrared heating technique.

Runs	Drying conditions		
	Infrared intensity (W/cm^2^)	Air temperature °C	Airflow (m/s)
1	0.10	35	0.3
2			0.5
3			1.0
4		45	0.3
5			0.5
6			1.0
7		55	0.3
8			0.5
9			1.0
10	0.20	35	0.3
11			0.5
12			1.0
13		45	0.3
14			0.5
15			1.0
16		55	0.3
17			0.5
18			1.0
19	0.40	35	0.3
20			0.5
21			1.0
22		45	0.3
23			0.5
24			1.0
25		55	0.3
26			0.5
27			1.0

### 2.4 Moisture content

Approximately 25 g of the sample was placed on a plate and dried in an oven set to 105 °C for 24 h to determine the okra's original moisture content (AOAC, 2005). The okra's initial moisture content was about 89.62 ± 0.5 % (wb). The okra's moisture content (MCdb) was determined using Eq. 1.

(1) $${\mathrm{MC}}_{\mathrm{db}}=\frac{W_{i}-W_{d}}{W_{d}}\;$$

The final moisture content (M_f_) was determined on dry bases using Eq.2.

(2) $$M_{f}=\frac{W_{\mathrm{wet}}-W_{d}}{W_{d}}\;$$

The Mt of the dry sample over time t is given by Eq. 3.

(3) $$M_{t}=\left[\frac{\left(M_{i}+1\right)\;W_{0}}{W_{t}}-1\right]=\frac{W_{t}-W_{d}}{W_{d}}\;$$

### 2.5 Drying kinetics

Using Fick's Law, a thin-layer drying model, the moisture ratio is determined as a function of drying time. The moisture ratio MR of the okra is calculated using equation 4, where $M_{t}$ is the moisture content at a given drying time (t), $M_{i}$ is the initial moisture content, and $M_{e}$ is the equilibrium moisture content.

(4) $$\mathrm{MR}=\frac{M_{t}-M_{e}}{M_{i}-M_{e}}\;$$

As stated, for infrared-convective drying, the equilibrium moisture content, $M_{e}$ was taken to be zero. Equation 5 shows the expression for the moisture ratio:

(5) $$\mathrm{MR}=\frac{M_{t}}{M_{i}}\;$$

### 2.6 Water activity

Okra samples (3 g) were used to evaluate water activity (a_w_) at 25 °C using the LabMASTER procedure. The machine measures the air humidity by enclosing the slices in a controlled, closed chamber to accurately and reliably assess water activity (aw).

### 2.7 Extract preparation

(Suri et al., 2022) outline the process for preparing the extracts with slight changes. 20 mL of 95% aqueous methanol is poured into a beaker to dissolve the finely ground, precisely measured 1.5 g of dried okra samples. After that, an extraction with ultrasound assistance is performed at 40 °C for 30 minutes. The supernatant is moved to a 50 mL centrifuge tube after standing for 30 minutes. The same procedure and time intervals are used to extract the leftover residue once more. The previous supernatant was mixed with the residue-derived supernatant. The extract was kept in the dark at -20 °C for quality analysis.

### 2.8 Total flavonoid content (TFC)

The TFC of the okra samples was measured using the procedure given by Dhurve et al. (Dhurve et al., 2022) with minor changes. First, 500 μL of okra extract was transferred to volumetric test tubes, and 250 μL of 5% NaNO₂ was added, followed by a 5-minute incubation. Afterward, 250 μL of 10% aluminium chloride was introduced, and after another 5 minutes, 2 mL of 1 M NaOH was added. The final volume was adjusted to 4000 μL by adding 1000 μL of 95% methanol. The solution was vortexed and incubated for a few minutes at room temperature in a dark place. Total flavonoid content (TFC) was then measured by recording absorbance at 510 nm and expressed as mg per gram.

### 2.9 Total phenolic content (TPC)

Using the FC reagent and standard curve established by (N. Jiang et al., 2017), the TPC of okra extract is calculated with a few changes. In brief, 100 μL of okra extract was placed into a test tube, and 500 μL of Folin-Ciocalteu (FC) reagent (diluted 1:10) was added. Next, 1500 μL of 7.5% Na₂CO₃ was added to the test tubes, followed by 1900 μL of distilled water to adjust the final volume to 4000 μL. The tubes were then incubated for 1 hour in a dark place. The absorbance was measured at 765 nm using a double-beam UV-VIS spectrophotometer (Shimadzu, UV-1800, Japan), with the blank as the reference. The calculation of the TPC was determined and expressed as mg/g.

### 2.10 Chlorophyll measurement

Green vegetables' ability to retain chlorophyll is a crucial indicator of product quality. The spectrophotometric (Shimadzu, UV-1800, Japan) technique was used to measure the amount of chlorophyll in the okra samples (Huang & Zhang, 2016). The filtrate was collected to calculate absorbance after dissolving the okra samples in 95% ethanol. The wavelengths at which chlorophyll "a" and "b" absorbance were measured were 665 nm and 649 nm, respectively. Using Eqs. (6), (7), the chlorophyll content in mg/kg was determined.

(6) $$\mathrm{Chlorophylla}=13.95A_{665}-6.88A_{649}$$

(7) $$\mathrm{Chlorophyllb}=24.96A_{649}-7.32A_{665}$$

### 2.11 Vitamin C

The vitamin C content of the sample was determined using the 2,6-dichlorophenol indophenol standard titration method (AOAC, 2005). The vitamin C analyses were performed in triplicates.

### 2.12 Rehydration ratio

Samples of dried okra were placed in boiling water to determine their rehydration properties. On a beaker filled with 150 ml of filtered water, the dried pods (10 g) were field hooked. Boiling continued for five minutes with the glass sealed (Doymaz & Kocayigit, 2012). Equation 8 was used to evaluate the dried sample's rehydration ratio (Rr).

(8) $$\mathrm{RR}=\frac{W_{r}}{W_{d}}\;$$

*W*_*d*_ is the weight of dried okra slices (g), and *W*_*r*_ is rehydrated okra slices (g).

### 2.13 Shrinkage ratio

The okra shrinkage ratio (Sr) was determined using a volumetric displacement technique, which involved measuring the water displaced by the samples. (Velić et al., 2007) established sample sizes and calculated average rates. The shrinkage ratio (Sr) was calculated using Equation 9.

(9) $$\mathrm{Sr}=1-\frac{V_{d}}{W_{o}}\;$$

where V_o_ is the volume of fresh okra, and V_d_ is the volume of dried slices.

### 2.14 Color analysis

The colour of fresh and dried samples was assessed with a colorimeter according to the method outlined by (Salehi & Kashaninejad, 2018). The five colour capacities were taken from the sample's surface for each examination, and the mean colour changes were verified. A white calibration plate was used to calibrate the measurement head. Following the standardization process, measurements were made of the fresh okra homogenate and the dried product powder's a* (red/green), b* (yellow/blue), and L* (brightness) values. At least ten measurements were made, and the average values were noted. Equations (10), (11) were then used to determine the browning index (BI) and total color difference (δE).

(10) $$\mathrm{\delta E}=\sqrt{{\left(L^{*}-L_{o}^{*}\right)}^{2}+{\left(a^{*}-a_{o}^{*}\right)}^{2}+{\left(b^{*}-b_{o}^{*}\right)}^{2}}\;$$

(11) $$\mathrm{BI}=100\times \left(\frac{x-0.31}{0.17}\;\right)\;$$

(12) $$X=\frac{a^{*}+1.75\;L^{*}}{\left(6.645\;L^{*}+a^{*}-3.012\;b^{*}\right)}\;$$

where a*, b*, L* are the colour parameters of dried okra slices, a_0_ *, b_0_ * and L_0_ *, are the colour parameters of fresh okra homogenate.

### 2.15 Texture analysis

A texture analyzer (TA-XT2i Stable Micro Systems Ltd, UK) was used to measure the hardness of the dried okra samples under the following parameters: probe P/2; test force using compression mode; RETURN TO START test; pre-experimental speed of 1.0 mm/s; experimental speed of 2 mm/s; post-experimental speed of 10 mm/s; compression 10%; trigger value/induction: Auto-5.0 g.

### 2.16 Machine learning approaches

#### 2.16.1 Artificial Neural Network (ANN)

**Figure 3:**
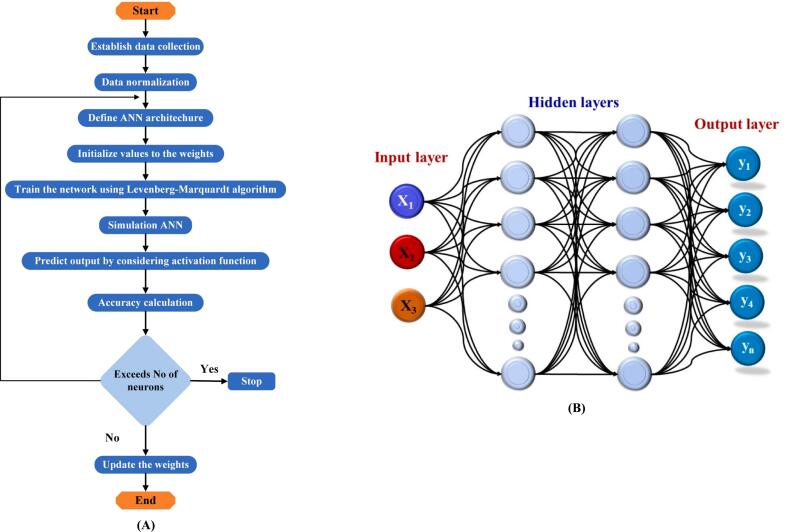
Structure of the Machine Learning using Artificial Neural Network (A) and flowchart of ANN with input and output data (B).

Artificial Neural Networks (ANNs) are a powerful, data-driven artificial intelligence technique. An ANN comprises three layers: input, hidden, and output. Data is transmitted via the neurons from an input layer to the layers below. The neurons in the output and input layers significantly impact the variables. Although having more neurons can reduce errors, it is also more time-consuming. Weights and transfer functions modify the signals passing through the neurons, and this process repeats until the desired output is achieved. The number of neurons in each layer can vary depending on the problem's structure. The ANN architecture in Figure 3 includes input, output, and hidden layers. The current ANN model comprises three input factors (T, AF, and IR) and okra physicochemical properties. The ANN model was successfully integrated with other methods, mainly the (SOM) approach, to optimize drying conditions and provide valuable data on a drying type.

#### 2.16.2 Self-organizing map (SOM)

The SOM procedure, a key example of unverified learning in neural networks, organizes varied data into a 2D feature map. The network architecture: a single layer applies these conditions to the neurons, which then successfully combine to form n × m clusters. Organizing these output parameters into 20×20 clusters resulted in 400 output neurons. Clustering was successfully executed using a method comparable to analytical techniques. This approach facilitates the creation of well-organized and understandable data groupings. Self-Organizing Map (SOM) is an unsupervised learning neural network that clusters variable data into a 2-D feature map with topological ordering based on their optimal similarity. The algorithm considers both the quality contents and the operating parameters as inputs. When this input pattern is fed to the network, the output layer units compete with each other, and when the output units come in closest weight with the income pattern, they are called the winners. Consequently, the network architecture of a single layer forwards these parameters to the winning output neurons known as neurons within n × m clusters. Thus, the architecture processes 9 input parameters arranged in a 20×20 cluster configuration, resulting in 400 output neurons (Fig. S1). The formation of groups is accomplished through seismic facies analysis, which detects various individual features and organizes them into coherent relational clusters. This technique identifies feature organizations within the dataset, enabling the creation of organized and meaningful clusters.

#### 2.16.3 Principal Component Analysis (PCA)

Principal Component Analysis (PCA), carried out with the Minitab 2022 LLC software, allowed for adding statistical details. This statistical technique tried deciphering the complex relations and differences between okra's physicochemical properties. The eigen-decomposition of the covariance matrix incorporates the anticipated data sets from the artificial neural network (ANN). Total variance and eigenvalue, important considerations for selecting and elucidating PCA, were used to pick the first two mechanisms (El-Mesery & Elabd, 2021). The PCA analysis of the governing parameters. Only the first two principal components, PC1 and PC2, are used to illustrate the correlations between the different quality metrics of okra slices since Kaiser-Guttman criteria only consider eigenvalues greater than unity.

#### 2.16.4 Validation methodology

The back-propagation technique, utilizing the sigmoid function, is employed during the ANN training stage. For the ANN model, private code was developed using MATLAB software. The dataset was randomly split, with 70% allocated for training and 30% for testing. The ANN setup with 12 neurons across two hidden layers offered the optimal balance between performance and regression accuracy. Back-propagation is a training method for ANN that remains stable at low learning rates. Additionally, all cases are subjected to the sigmoid function, as described in Eq. 13:

(13) $$f\left(x\right)=\frac{1}{1+e^{-x}}\;$$

Before being integrated into the ANN framework, the data set was adjusted according to Eq. 14:

(14) $$X_{i}=\frac{x_{i,\max}-x_{i}}{x_{i,\max}-x_{i,\min}}\;$$

### 2.17 Statistical Analysis

The models' performance was estimated using statistical metrics such as correlation coefficient (R^2^) and root mean square error (RMSE). The R^2^ and RMSE are defined by equations (15), (16).

(15) $$R^{2}=\frac{\sum_{i=1}^{N}{\left({\mathrm{MR}}_{\exp.i}-{\mathrm{MR}}_{\mathrm{Pre}.i}\right)}^{2}}{\sqrt{\left[\sum_{i=1}^{N}{\left({\mathrm{MR}}_{\exp.i}-{\mathrm{MR}}_{\mathrm{Pre}.i}\right)}^{2}\right]*\left[\sum_{i=1}^{N}{\left({\mathrm{MR}}_{\exp.i}-{\mathrm{MR}}_{\mathrm{Pre}.i}\right)}^{2}\right]}}\;$$

(16) $$\text{RMSE}=\sqrt{\frac{1}{n}\sum_{i=1}^{n}{\left(X_{m}-X_{p}\right)}^{2}}\;$$

Triplicate experiments were performed for each drying condition. The experimental data are presented as means ± standard deviations (SD). Table S1 shows the performance of the ANN and the regression factor across 1 to 3 hidden layers and 4 to 14 neurons. The SPSS statistics software (version 27.0, SPSS Inc., Chicago, IL, USA) was used for statistical analyses. The effects of various operating conditions on the drying properties and quality parameters were assessed using an analysis of variance (ANOVA), followed by Duncan's multiple range test as a post hoc analysis, with a significance level of 0.05.

## 3 Results and discussions

### 3.1 Drying kinetics

**Figure 4:**
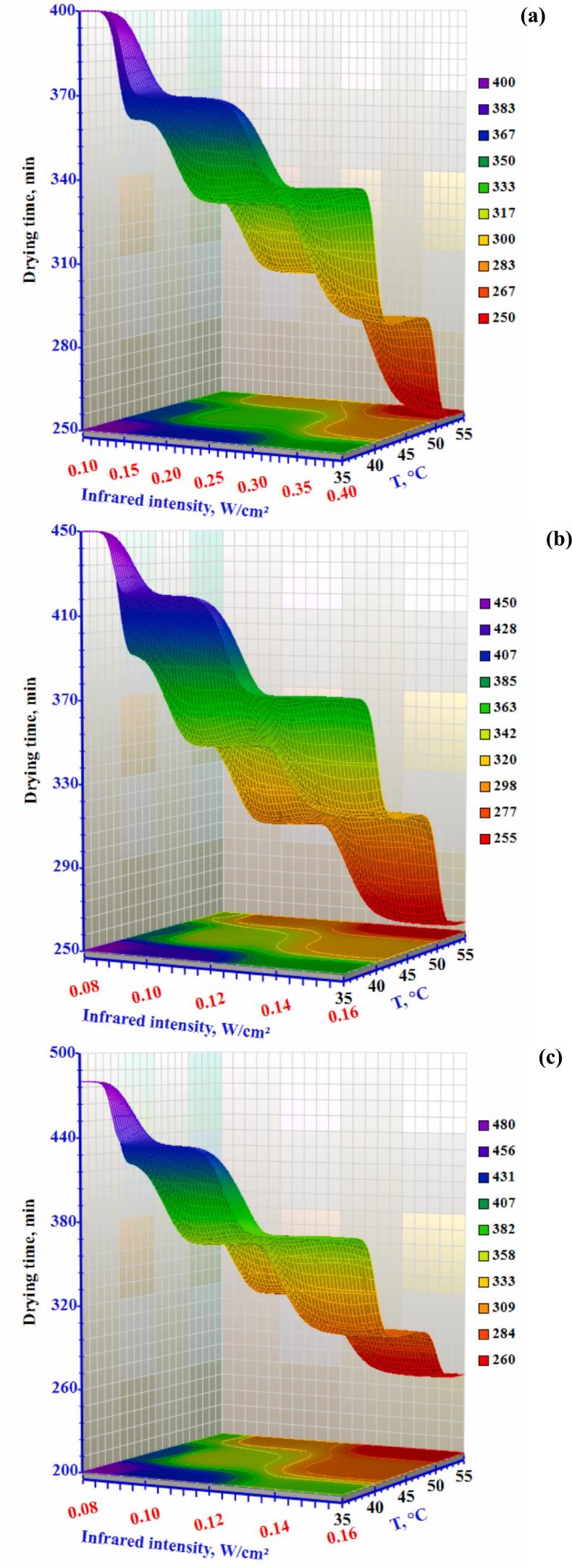
Effect of infrared radiation intensity and air temperature on the drying time of okra slices at airflow rates of (a) 0.3 m/s, (b) 0.5 m/s, and (c) 1.0 m/s.

Figure. 4 depicts the air temperature, air velocity, and infrared intensity effect on the okra slices' drying characteristic curves during combination drying. Drying time reduces when air temperature and radiation intensity increase. The okra slices' elevated temperature and enhanced radiation intensity are the reasons for this phenomenon. (An et al., 2024) observed that increasing the intensity of infrared radiation can increase the drying rate of food and reduce the drying time. This increase in radiation intensity produces a greater temperature difference on the surface or lower slices of the product (Table S3), which accelerates the water evaporation rate and shortens the required drying time. Under conditions of 35°C temperature, 0.3 m/s airflow speed, and infrared intensities of 0.10, 0.20, and 0.40 W/cm^2^, the drying times needed to reduce the moisture content from an initial value of 89% (wb) to a final moisture content of 10% (wb) were 400, 370, and 340 minutes, respectively. When the air temperature increased from 35°C to 55°C while maintaining the intensity level of the infrared at 0.10 W/cm^2^ and the wind speed at 0.3 m/s, the drying time reduced to 250 min. Similar drying trends were observed for okra slices at different drying air temperatures and airflow speed levels. Under all test conditions, with IR intensity and air temperature held constant, an increase in airflow speed resulted in longer drying time, which is likey due to an enhanced cooling effect on the product surface, which reduces the surface temperature (Du et al., 2023). In general, the time required to reduce the moisture content to any given value depends on the drying conditions, being highest at IR intensity of 0.10 W/cm^2^, 35°C and 1.0 m/s, and lowest at IR intensity of 0.40 W/cm^2^, air temperature of 55°C and air speed of 0.3 m/s.

### 3.2 Water activity (a_w_)

**Figure 5:**
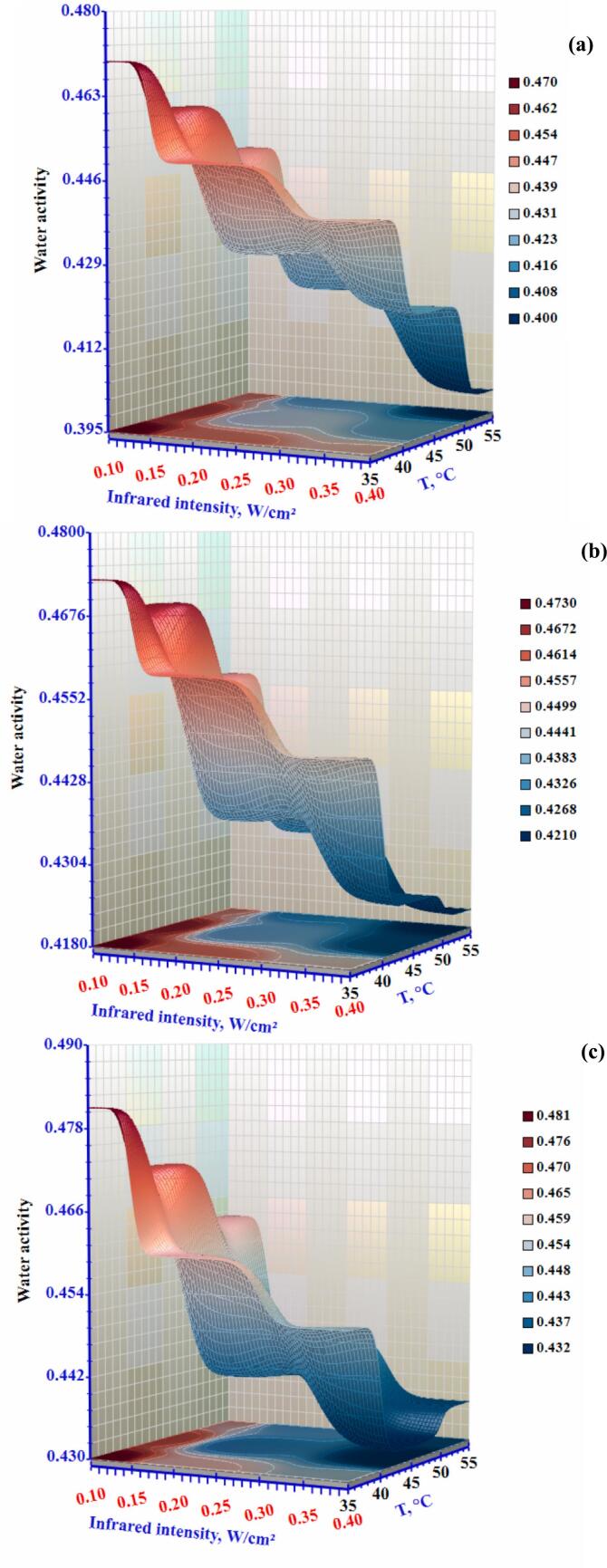
Effect of infrared intensity and air temperature on the water activity of okra slices dried using an infrared-convective conveyor belt dryer at airflow rates of (a) 0.3 m/s, (b) 0.5 m/s, and (c) 1.0 m/s.

Low water activity foods are defined as having a water activity (a_w_) level below 0.8. For dry products, the required water activity is typically 0.6, which is the threshold below which yeast and microbial reproduction is inhibited. (Dhaliwal et al., 2021). Figure 5 displays the (a_w_) of the slices as a function of different drying settings. The (a_w_) of okra is lower than 0.6 in all tests, signifying that the slices are free from archetypal bacterial harm. The (a_w_) values for the sample ranged from 0.43 to 0.48. The faster moisture evaporation from the sample is attributed to the combined effects of increasing infrared radiation and air temperature, along with decreasing airflow. (Wang & Chao, 2002). The faster attainment of the required water activity (a_w_) is facilitated by increased water loss from the slice and more active moisture diffusion within the slices (Royen et al., 2020). The findings of this investigation show that drying using an infrared heating process can offer reliable (a_w_) values for extended shelf life for the okra.

### 3.3 Vitamin C content

**Figure 6:**
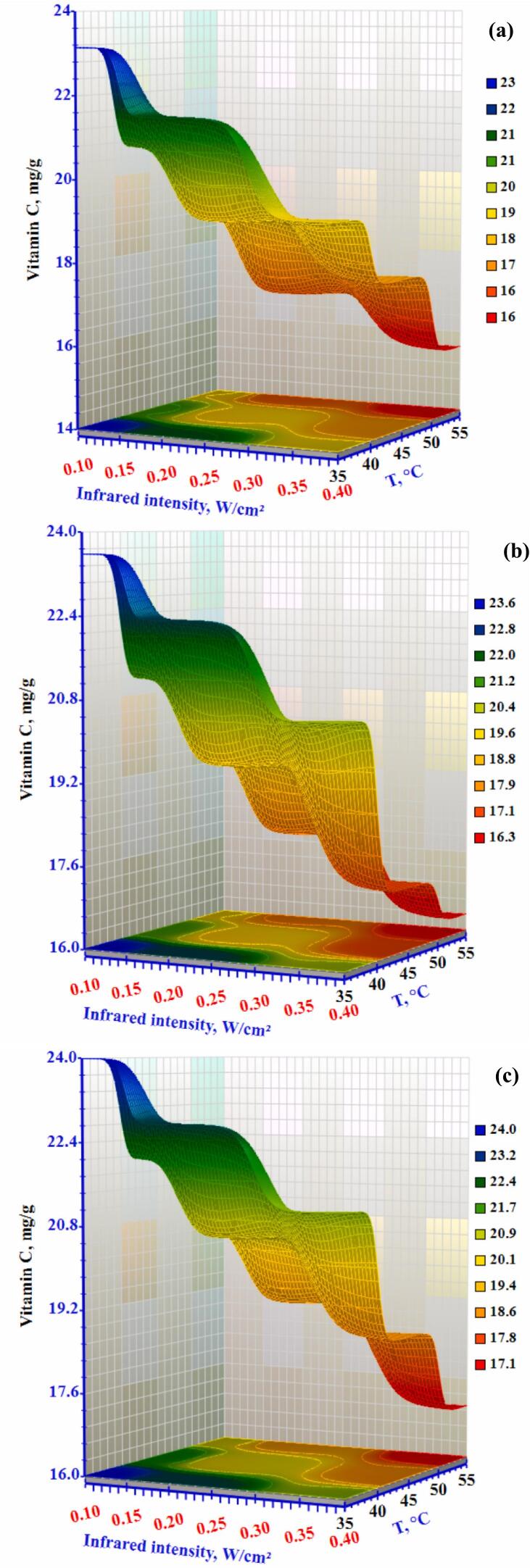
Effect of infrared intensity and air temperature on the Vitamin C content of okra slices at airflow rates of (a) 0.3 m/s, (b) 0.5 m/s, and (c) 1.0 m/s.

Vitamin C can be found in most fresh fruits and vegetables; however, it is not very stable and is usually degraded during processing when subjected to heat, oxygen, or light (Demircan et al., 2024). The influence of varying infrared radiation, air temperatures, and airflow on the Vitamin C of the dried okra slice is shown in Figure 6. As air temperature and radiation intensity increase, vitamin C in dried okra slices reduces. The minimum vitamin C content, 15.62 mg/g, was observed at a drying temperature of 55 °C, air velocity of 1.0 m/s, and infrared intensity of 0.4 W/cm^2^. The maximum vitamin C content, 23.99 mg/g, was recorded at 35 °C, 0.3 m/s air velocity, and 0.10 W/cm^2^ infrared intensity. Due to its heat sensitivity, vitamin C reduces rapidly under high temperatures. To preserve vitamin C while achieving efficient drying (Table S3), a short drying time using low-temperature methods is recommended. It will break down by increasing the duration of the dehydrating process. The lowest level of vitamin C was recorded at higher air temperature and infrared intensity. The slice's additional time in the dryer and higher infrared intensity, which ultimately changed the composition of the finished good and caused thermal degradation, is responsible for the drop in vitamin C. (Li et al., 2019) examined the improvement of the conditions for drying okra using different drying methods. Research on hybrid dryer revealed that extending the airflow from 0.3 to 1.0 m/s while maintaining constant air temperature and power levels extended the dehydrating time. Instead of decreasing as might be expected during hot air drying, the drying time increased. Contrary to expectations for hot air drying, the drying time increased, likely due to the cooling effect caused by higher air velocity, which lowered the temperature of the slices. However, at an airflow of 2.0 m/s, 40 W power, and 60 °C, the dried sample exhibited the highest vitamin C content during the drying process (Sharma & Prasad, 2006).

### 3.4 Total phenolic content (TPC)

**Figure 7:**
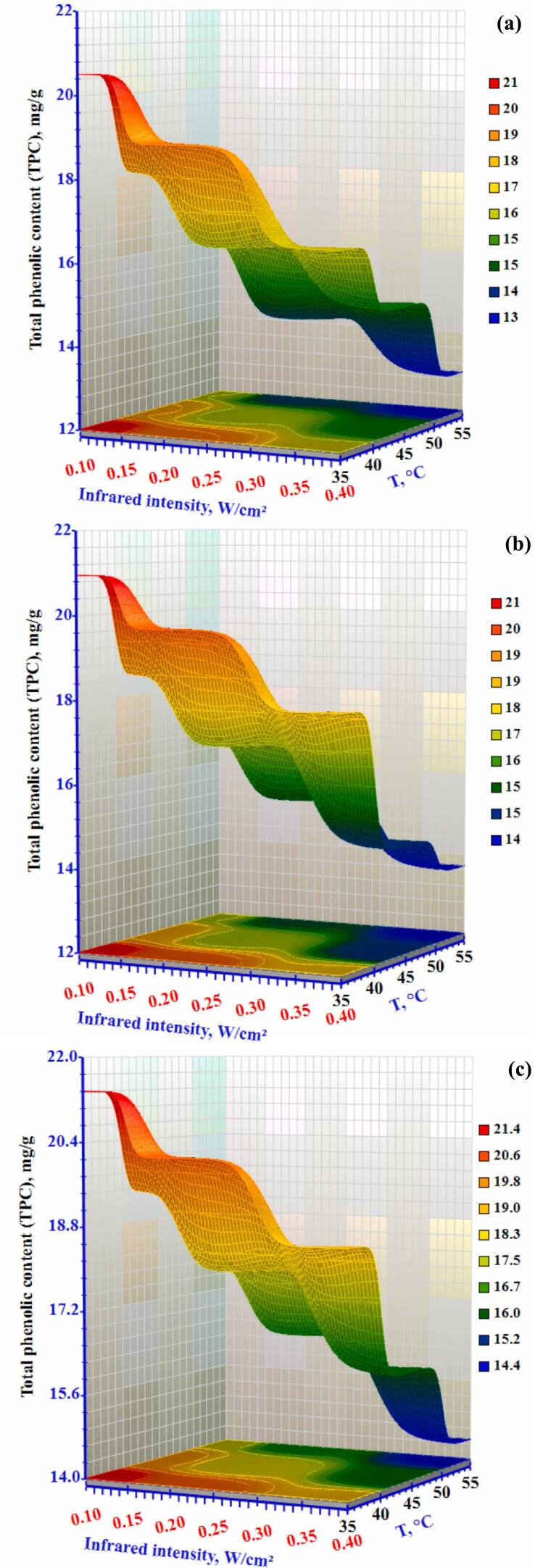
Effect of infrared intensity and air temperature on the total phenolic content (TPC) of okra slices dried using an infrared-convective conveyor belt dryer at airflow rates of (a) 0.3 m/s, (b) 0.5 m/s, and (c) 1.0 m/s.

Total phenolic compounds (TPC), usually secondary metabolites, serve diverse therapeutic and physiological activities in living organisms and are extremely important in scavenging free radicals (Xu et al., 2020). The fresh okra's TPC value is 15.05 mg/g. As shown in Figure 7, the TPC concentration at 1.0 m/s under 0.4 W/cm^2^ was 14.42 mg/g, with the lowest value recorded at a temperature of 55 °C. The air temperature and velocity were 35 °C and 0.5 m/s, respectively, and the highest (TPC) concentration was 20.51 mg/g under 0.10 W/cm^2^. The TPC increased significantly (Table S3) by 1.51 and 2.53 times after drying at 35 °C and 55 °C, respectively. However, the TPC increased and then decreased with higher drying temperatures. This could be because phenolic compounds would degrade due to the high drying temperature and the extended drying period. In general, when the drying temperature increased, the TPC also increased. This may be attributed to the higher drying temperatures, which shorten the drying period and reduce the thermal degradation of phenolic compounds. Environmental factors, such as drying temperature during the process and the applied processing techniques, have been shown to influence TPC levels significantly. A similar outcome was noted when okra was dried by hot air (H. Li et al., 2019). The TPC value increased until it reached 70 °C and decreased as the drying temperature increased. Processing methods, such as drying temperature and infrared radiation during the drying process, may affect TPC, per earlier research.

### 3.5 Total flavonoid content (TFC)

**Figure 8:**
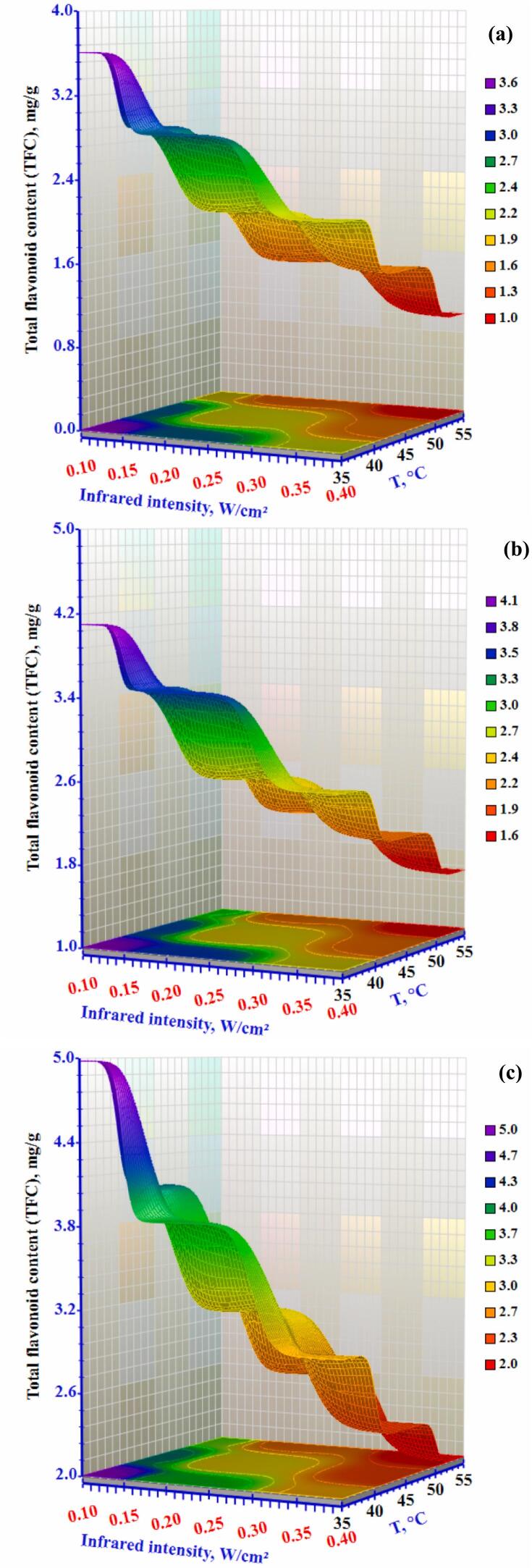
Effect of infrared intensity and air temperature on the total flavonoid content (TFC) of okra slices dried using an infrared-convective conveyor belt dryer at airflow rates of (a) 0.3 m/s, (b) 0.5 m/s, and (c) 1.0 m/s.

Figure 8 displays the total flavonoid content (TFC) in methanol extracts from dried okra slices. The fresh okra sample has a TFC of 10.71 ± 0.03 mg/g, which decreased under various drying conditions. The fresh sample's TFC was significantly higher than the control sample's (p < 0.05). Using hybrid infrared drying, the TFC ranged from 0.99 to 4.98 mg/g. The highest TFC, 4.98 mg/g, was achieved with an infrared intensity of 0.10 W/cm^2^, air temperature of 35 °C, and airflow rate of 1.0 m/s. TFC values generally decreased as infrared intensities and air temperatures varied with changes in airflow rates. The retention of TFC at high temperatures with low airflow rates may result from the formation of new flavonoid compounds due to the breakdown of cell structures in Maillard reactions under high temperatures (İzli et al., 2022). Additionally, lower airflow may help sustain higher temperatures and reduce drying time, preserving volatile and bioactive compounds more effectively.

### 3.6 Chlorophyll measurement

**Figure 9:**
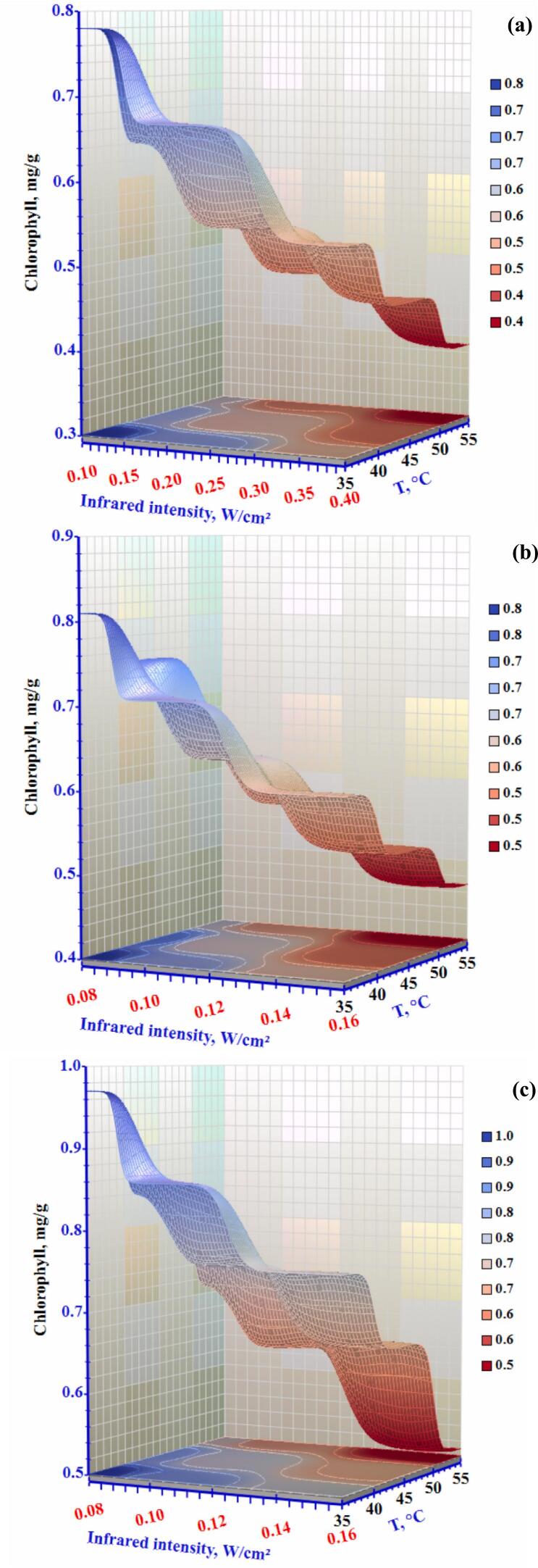
Effect of infrared intensity and air temperature on the Chlorophyll content of okra slices dried using an infrared-convective conveyor belt dryer at airflow rates of (a) 0.3 m/s, (b) 0.5 m/s, and (c) 1.0 m/s.

One crucial factor influencing the color of dried goods is their chlorophyll content. The drying process significantly influenced Chlorophyll breakdown during drying, which led to color alterations in the finished goods. The primary elements influencing chlorophyll breakdown are enzymes, light, and oxygen (Ledari et al., 2024). Figure 9 illustrates the correlation between the chlorophyll content and colour values influenced by drying settings. Thus, it may be concluded that the composition of green okra was mostly determined by its chlorophyll level. An efficient way to increase chlorophyll retention was to balance low temperature and a short period. A similar phenomenon has been observed in previous research on toona sinensis drying (H. Li et al., 2019). When okra was dried at several temperatures, its chlorophyll concentration ranged from 1.11 to 1.66 mg/g db and decreased by around 9.77% when the temperature rose from 55 to 70 °C. The hot water treatment also considerably impacted the breakdown of chlorophyll content. There was a greater loss of total chlorophyll when the temperature increased from 70 °C to 90 °C. Because chlorophyll is heat-sensitive, it may have leached away due to thermal destruction during treatment. (Xu et al., 2021) also noticed a reduction of chlorophyll during ultrasonic pre-treatment.

### 3.7 Rehydration ratio

**Figure 10:**
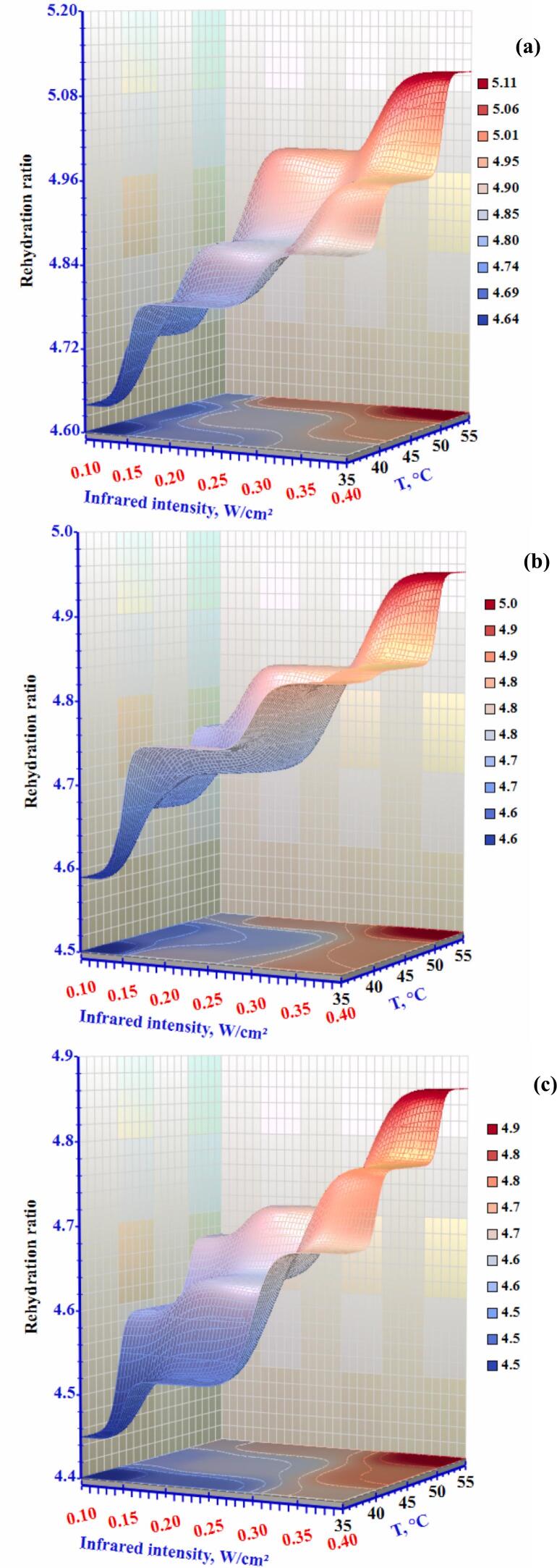
Effect of infrared intensity and air temperature on the rehydration ratio of okra slices at airflow rates of (a) 0.3 m/s, (b) 0.5 m/s, and (c) 1.0 m/s.

For dried food products, the rehydration ratio is a critical metric. The complex rehydration process is influenced by the therapies used before dehydration and the chemical and physical changes that occur during drying. The rehydration ratio is a qualitative parameter that shows the ability of food to return to its original form after drying and the amount of cell destruction during drying (Leiton-Ramírez et al., 2020). Variations in the rehydration ratio of okra slices after the drying process were measured according to the method mentioned in Section 2.12 and Eq. 8. According to Figure 10, the combination of an infrared intensity of 0.10 W/cm^2^, an air temperature of 35 °C, and airflow rate of 1.0 m/s resulted in the least rehydration of 4.45. The results showed that velocity has no significant effect on the rehydration of okra slices. Minimal variations in rehydration were experiential when the air temperature was 55 °C and the infrared intensity was 0.4 W/cm^2^. The rehydrated ratio of dried okra varied between 4.45-4.87 for all the drying conditions. It was observed that the rehydration ratio increased with high infrared and low airflow. The rehydration ratio increased with higher radiation intensity and air temperature as the greater infrared energy generated more porous structures in the dried samples. (El-Mesery et al., 2023) also reported that IR improved rehydration by reducing the drying time. Consequently, IR exposure on the slice surfaces created mini-channels, which later became pores, facilitating more efficient water transmission to the samples. Additionally, the rehydration ratio increased with rising air temperatures. This suggests that the air temperature may cause variations in the product, potentially leading to a loss of solids during rehydration, thereby facilitating the rehydration process. This effect arises from the increased IR, which raises the air velocity around the slices. Consequently, there is an increase in the temperature gradient over the underlying slices or surface layer, accelerating the rate of moisture evaporation. Additionally, the rehydration rate of okra improves with higher drying temperatures. At higher temperatures, the food's rehydration rate increases because of the temperature's effect on tissue collapse and cell damage.

### 3.8 Shrinkage ratio

**Figure 11:**
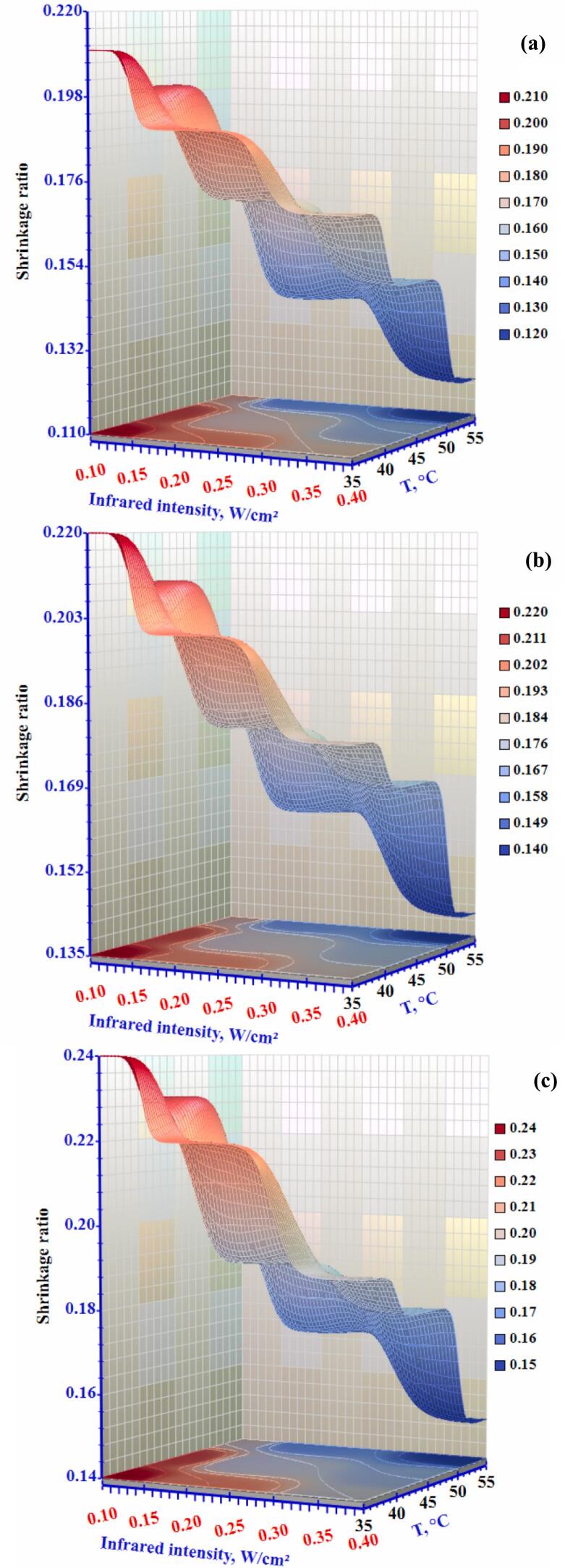
Effect of infrared intensity and air temperature on the shrinkage ratio of okra slices at airflow rates of (a) 0.3 m/s, (b) 0.5 m/s, and (c) 1.0 m/s.

Foodstuff shrinkage is a normal physical occurrence seen throughout various drying techniques. It affects the quality of the dehydrated products. It should be considered when predicting moisture and temperature profiles in the dried material (Li et al., 2024). Figure 11 illustrates the shrinkage ratio of okra slices when dried under a combination of convective and infrared conditions. The shrinkage ratio decreased from a high value of 0.21 to a low value of 0.17 when the radiation intensity was increased from 0.10 to 0.40 W/cm^2^ while maintaining a constant airflow of 0.3 m/s. Conversely, the shrinkage ratio reduced from 0.24 to 0.19 with the same radiation intensity setting at a drying air velocity of 1.0 m/s. This reduction is consistent with the typical shrinkage observed in samples due to dehydration during drying. Removing moisture from tissues creates a pressure differential between the tissue's interior and outside, which leads to compressive stresses and shrinking (El-Mesery & Elabd, 2021). Therefore, the lowest shrinkage ratio values were obtained when operating at high infrared radiation intensity and moderate airflow. Low shrinkage under increased infrared intensity and low airflow can be ascribed to higher product temperatures and the lower moisture content of the exterior surfaces on both sides of the slice.

### 3.9 Colour attributes

**Figure 12:**
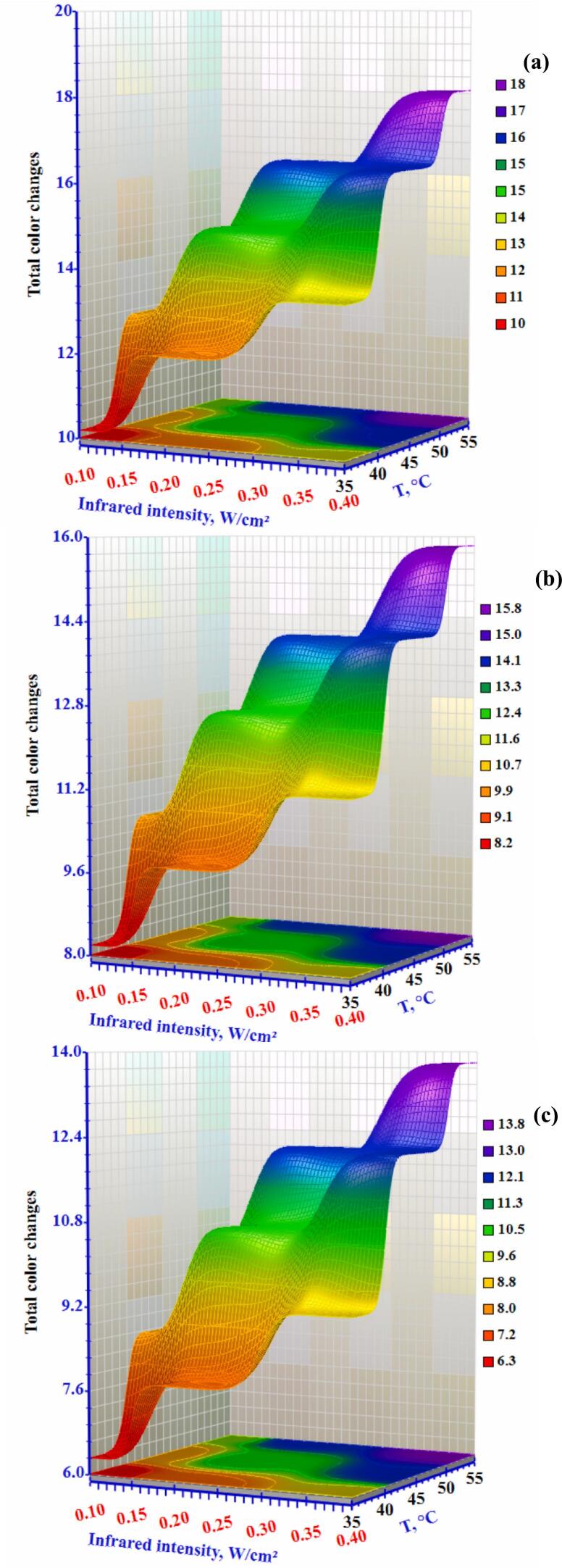
Effect of infrared intensity and air temperature on the drying conditions of okra slices at airflow rates of (a) 0.3 m/s, (b) 0.5 m/s, and (c) 1.0 m/s.

One of the most crucial factors in determining the quality of dried okra slices is their colour. One important quality factor influencing customer acceptance and the market value of foods is colour. The lowest δE is used as the manufacturing standard for assessing the colour of dry okra. The results of the okra slices' total colour change are represented in Figure 12. Increasing the infrared from 0.10 to 0.40 W/cm^2^ at 0.3 m/s caused the δE to increase from 10.2 to 13.52. The δE increased from 15.36 to 20.08 at an air temperature of 35 °C with the same infrared intensity range and airflow of 1.0 m/s. Statistical analysis showed that the δE of dried okra was considerably impacted by the drying air temperature and infrared intensity, with δE increasing remarkably as temperature and infrared intensity levels increased. As the drying temperature increased, the δE increased in each treatment due to more severe non-enzymatic browning at higher temperatures. This finding aligns with (Geng et al., 2022), who found that extended dehydration processes lead to more pronounced browning reactions. These reactions affect the compounds in the samples and likely contribute to pigment degradation as well as enzymatic and non-enzymatic reactions. The δE increased with higher infrared intensity but decreased with greater airflow. The decrease in δE with increased airflow can be credited to the cooling influence of the passing airflow on the okra slices within the dryer, resulting in overall heat loss to the drying chamber. Taghinezhad et al. (Taghinezhad et al., 2021) investigated the overall colour change in organic blackberries during hybrid hot air/infrared drying, both with and without ultrasonic pretreatment, for 15, 30, and 45 minutes. According to their findings, the samples' overall colour variance consistently decreased as the drying air temperature increased from 50 to 60°C and then from 60 to 70°C. Similarly, Özbek (Özbek, 2021) examined the overall sweet potato colour change using hybrid infrared/hot air drying. The study found that higher air temperatures led to smaller changes in the colour of samples with a thickness of 6 mm.

**Figure 13:**
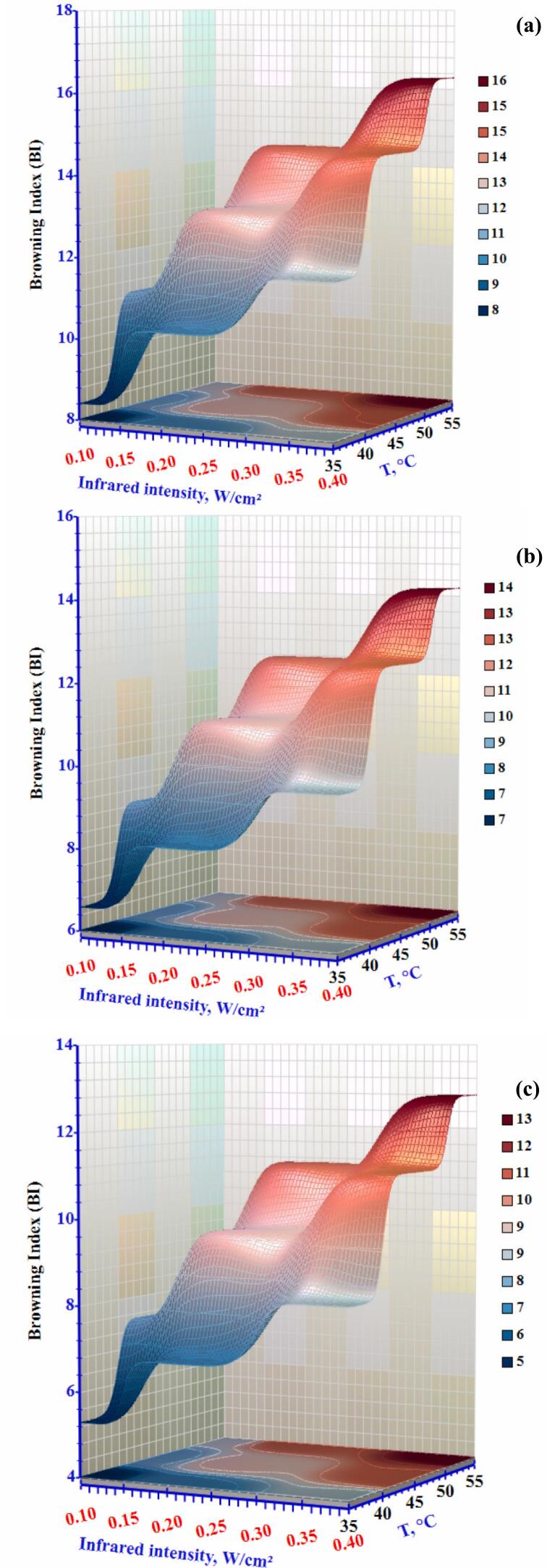
Effect of infrared intensity and air temperature on the browning index of okra slices at airflow rates of (a) 0.3 m/s, (b) 0.5 m/s, and (c) 1.0 m/s.

Browning Index (BI) was used to evaluate the browning degree resulting from enzymatic and non-enzymatic reactions. A drop in the Browning Index (BI) was seen as the drying temperature increased from 35°C to 55°C (Fig. 13). When the temperature was increased from 35 °C to 55 °C, the BI showed a significant reduction of 19.51% and 17.13 %, respectively. Higher temperatures promote both enzymatic and nonenzymatic browning (Maillard reactions), which cause changes in the colour of food materials during drying.

### 3.10 Texture analysis

**Figure 14:**
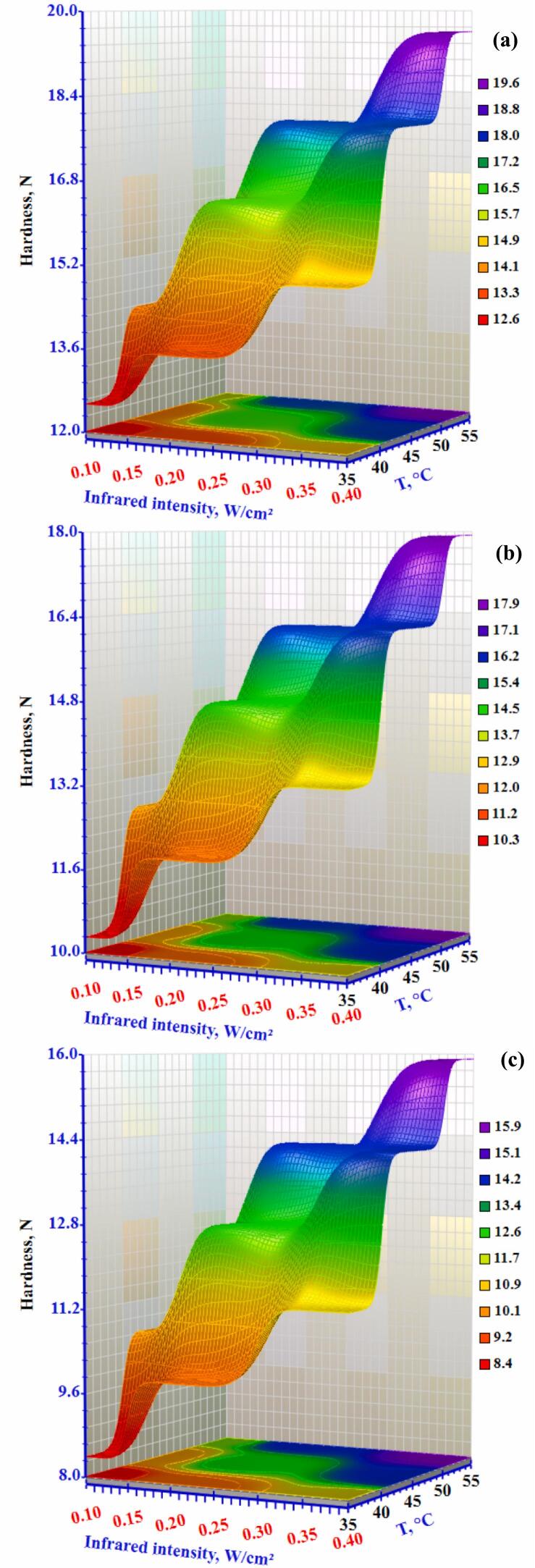
Effect of infrared intensity and air temperature on the hardness of okra slices at airflow rates of (a) 0.3 m/s, (b) 0.5 m/s, and (c) 1.0 m/s.

Texture is one of the most significant indicators of dried product quality, especially hardness. It describes how the senses perceive a food's structure and respond to force (Xu et al., 2021). Okra is typically assessed using two criteria: frangibility and hardness. The test can only be conducted when the sample's water activity is less than 0.4. Hardness is an index used to assess the material's softness and hardness. Figure 14 clearly show that the drying conditions substantially impacted the hardness (p < 0.05) of the dried okra's texture. At an airflow of 0.3 m/s and temperature of 35 °C, the hardness of dry okra slices increased significantly from 12.55 to 15.04 N. Conversely, the dryness of the okra slices decreases from 8.43 to 11.37 N at 1.0 m/s as the infrared intensity increased from 0.10 to 0.40 W/cm^2^. The infrared intensity and air temperature regression coefficients were significant (p≤0.05), while the airflow coefficient was negligible. The dried okra's hardness also increased as the air temperature increased. Other dried materials showed considerable surface shrinkage and structural collapse due to high-temperature heating operations, which increased their hardness. This observation aligns with the findings of Aradwad et al. (Aradwad et al., 2023).

### 3.11 Machine Learning Prediction

**Figure 15:**
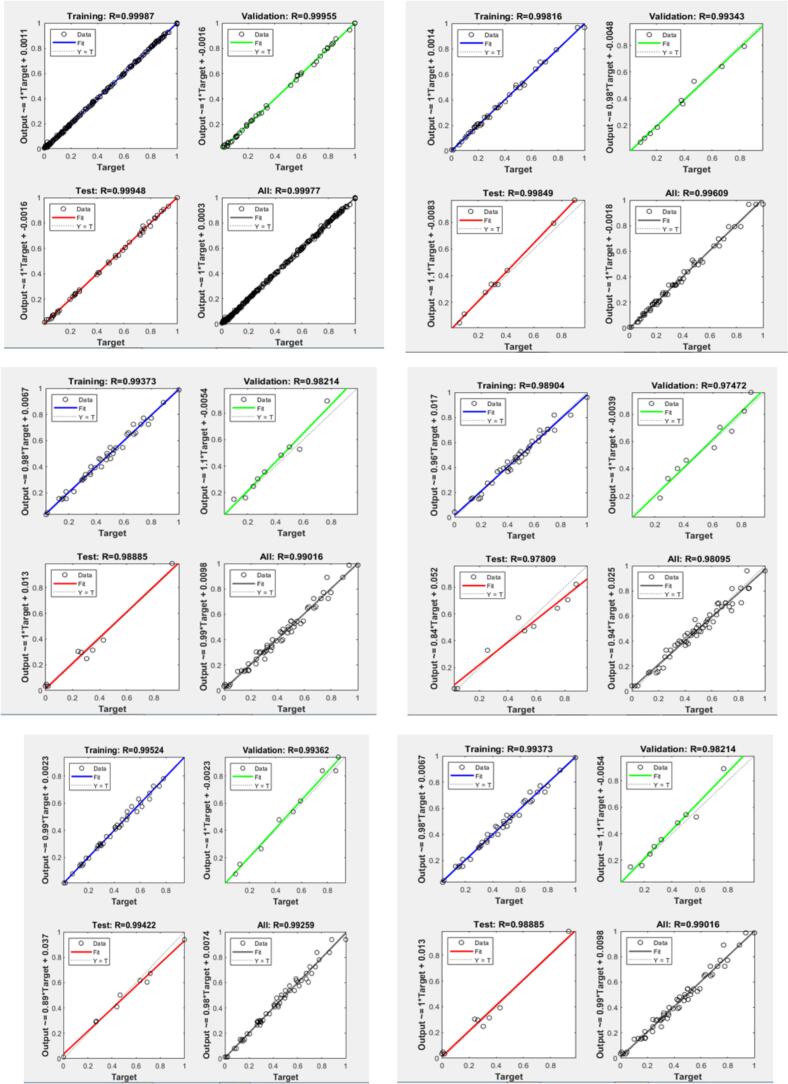
Performance of the Machine Learning using Artificial Neural Networks (ANNs) model during training, testing, and validation.

Machine Learning modelling (ML) model was used in this investigation to forecast different parameters related to okra drying. In many different configurations, the Artificial Neural Network (ANN) is the ideal method for predicting the results of non-linear structures. The key components of the procedure, as outlined by Kutyauripo et al. (Kutyauripo et al., 2023), include estimating the output data for the desired parameter combinations, overlaying the technique's behavior, and populating the network with empirical data. The effect of neuron counts, occupation categories, and learning procedures on prediction accuracy was evaluated by testing various network configurations using a trial-and-error method for network structure selection and simulation. Figure 15 illustrates how the performance of the ANN archetypal was calculated by contrasting predicted output data. Radiation, airflow, and temperature play essential roles in controlling the quality attributes of all variables. Radiation and airflow primarily influence the data for rehydration, while temperature is a second priority. Radiation was the first factor influencing colour, and water activity, followed by temperature and airflow. The interaction between factors is taken into account, unlike a single-factor experiment, which cannot detect interactions among parameters, particularly concerning colour. The airflow contact is more significant for TFC, TPC, and vitamin C, but the radiation interaction is more important for colour, brewing index, water activity, hardness, rehydration, and shrinkage ratio.

**Figure 16:**
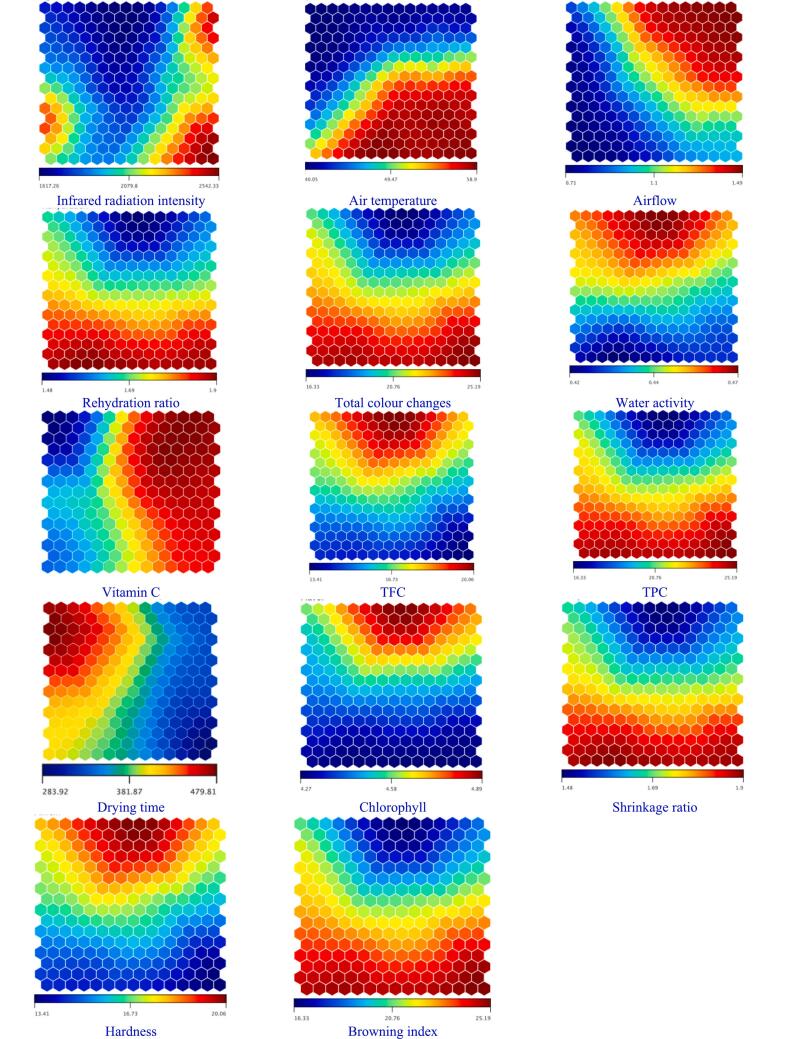
The SOM analysis of the current investigation involves.

The ANN simulations are combined using extra algorithms to improve dryer settings or monitor the dehydrating technique through the self-organizing map (SOM) method. Although the previous evaluation provides an extensive overview of the quality material, self-organizing maps (SOM), an exciting information extraction approach, can provide an alternate knowledge of the best working conditions. The SOM representations are shown in Figure 16, which are grouped into four clusters according to feature similarity. Significant differences in air flow, temperature, and infrared intensity across all clusters can be observed on the map in Figure 16, providing substantial context for our study. The map displays high water activity, rehydration, colour, and low vitamin C for the first cluster. This cluster has higher values of both convection and radiation. The third cluster's water activity exhibits medium values, while rehydration shows reduced values. The colour indicates relatively low-to-medium values, while the vitamin C shows medium-to-high values. This cluster has a wide range of temperature and radiation changes, from low to high values. The second cluster's water activity exhibits lower values, while the rehydration and colour show medium values. This cluster has high-airflow values with medium values for both temperature and radiation. In the fourth cluster, all variables show higher values, whereas both convection and radiation also have higher values.

**Figure 17:**
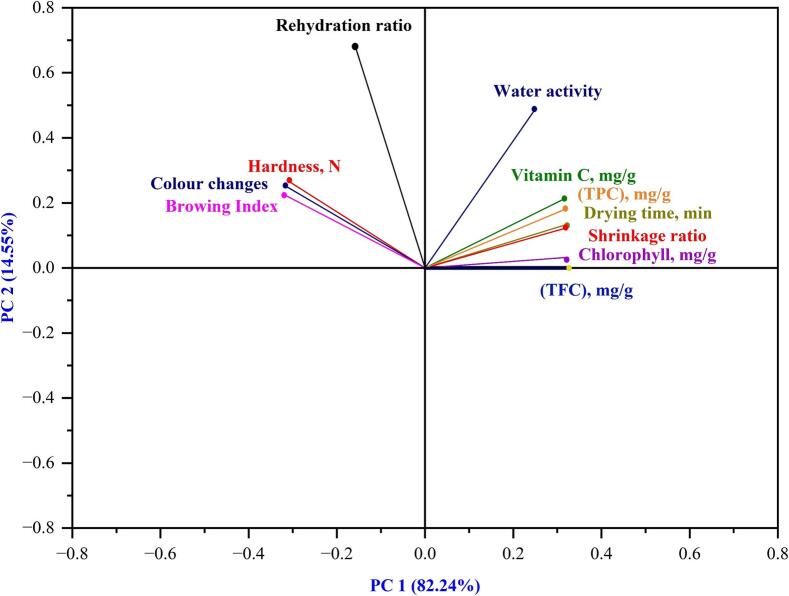
Principal component analysis (PCA) on the drying process.

To evaluate differences between various okra, slice drying conditions and identify correlations with specific components, the (PCA) statistical tool was used, retaining a significant portion of the original data. Figure 17 shows the performance of the PCA drying durations under various circumstances. With PC1 and PC2 accounting for 82.24 % and 14.55 % of the variance, respectively, the cumulative explained variance was 82 percent, falling between 70 % and 90 %, the recommended range for sufficient variability explanation (Wahia et al., 2020). According to the eigenvectors, each variable affects PC1's structure reasonably, with the vitamin C variable having the most significant impact on all of the parameters in this component's composition. Vitamin C and water activity correlate positively, indicating an increase in them. The water activity, vitamin C, TPC, TFC, drying time, shrinkage ratio, and Chlotphy are positive PC2 and PC1. Accordingly, the accompanying information shows that while drying harms colour and rehydration, it has a good impact on flavour, water activity, vitamin C, and TPC.

The principal component analysis (PCA) and Self-Organizing Map (SOM) highlight the relationships among variables and offer insights into the key factors influencing variability in data collection. Applying the results from the SOM, industrial drying processes can be optimized to achieve a balance among product quality, energy efficiency, and environmental sustainability. These improvements will result in increased consistency and quality of dried products, lower operational costs, and compliance with contemporary requirements for sustainable food production systems. The SOM and PCA analyses provide valuable insights into the effects of critical drying parameters, including infrared radiation intensity, air temperature, and airflow, on the quality of the product. This information may be utilized to optimize industrial drying systems. Establish real-time oversight and regulation of drying parameters. Industries can use AI-driven systems to dynamically adjust drying conditions, ensuring they remain within the optimal regions identified in the SOM lower air temperature in the later drying stages to decrease nutrient degradation and minimize the browning index. The SOM demonstrates that drying conditions preserve elevated concentrations of bioactive compounds.

Energy efficiency represents a significant issue in industrial drying processes. The SOM analysis assists industries in minimizing energy consumption while preserving product quality. The SOM regions indicating decreased drying time can assist in identifying energy-efficient combinations of infrared intensity, airflow, and temperature. Translating SOM insights from laboratory-scale to industrial-scale processes necessitates meticulous validation and execution. The SOM facilitates consistency in production batches by identifying stable zones for quality attributes. This minimizes variability in product quality in large-scale industrial operations.

## 4 Conclusion

The application of machine learning algorithms to optimize food drying processes is gaining popularity in the food industry, as these techniques improve drying efficiency while preserving phytochemical content and physicochemical properties. This investigation examined how various airflow, temperature, and infrared intensities affect okra slices' phytochemical content and physicochemical properties. The following conclusions were drawn from the study:

1- The drying time for dry okra slices increased by increasing the airflow, while it decreased by increasing the IR intensity and air temperature.
2- The total colour difference and the rehydration ratio increased with higher IR intensity and air temperature but decreased with lower airflow.
3- The Artificial Neural Network (ANN) model provided accurate predictions that aligned well with the testing data sets, offering valuable insights into understanding and controlling the factors affecting the okra drying process.
4- Optimal drying conditions for infrared drying of okra were identified at airflow of 0.3 m/s, air temperature of 55 °C, and infrared intensity of 0.40 W/cm^2^.
5- The study highlights the importance of combining advanced statistical analysis and machine learning to optimize foodstuff processing.

The results of this study have important practical implications for food processing industries, especially those involved in drying fruits and vegetables. The optimization of drying conditions, such as airflow, temperature, and infrared intensity, can help achieve superior dried food products with enhanced phytochemical retention and minimal physicochemical degradation.

## 5 Suggestions for Future Research

Future research could expand on this study by exploring the use of machine learning models and advanced statistical techniques across a broader range of food types, including fruits, vegetables, and herbs. Comparative studies on different drying methods (e.g., freeze-drying, hot air drying, and microwave drying) could offer a more holistic view of optimal processing conditions for various food materials. Investigating the combined effects of drying parameters on the sensory quality (taste, aroma) and shelf-life stability of dried products would also be beneficial. Future studies could also investigate the environmental impacts of these drying techniques, exploring the potential for energy savings and reduced carbon footprints. Lastly, investigating consumer preferences for dried food products processed under optimal conditions could guide industry practices and ensure that the developed methods align with market demands.

## 6 The limitations of the proposed study

A limitation of the study lies in the possible bias in the data collection process, primarily if the data is sourced from a single batch or limited geographical location. The okra slices' phytochemical content and physicochemical properties can vary based on growing conditions, harvest time, and cultivar differences. If the data is not sufficiently diverse, it could lead to overfitting models to specific conditions, thus limiting their applicability to a wider range of environments or production methods. Although the study focuses on okra, the proposed machine-learning model may not directly apply to other vegetables with different biochemical and physicochemical properties. Further research would be required to determine the generalizability of the method to a broader range of crops, requiring adjustments to the model or data collection techniques. : The study may have limited long-term validation, especially regarding preserving phytochemicals and physicochemical properties over extended storage periods or under various processing conditions.

## CRediT authorship contribution statement

**Hany S. El-Mesery:** Writing – review & editing, Writing – original draft, Visualization, Supervision, Software, Methodology, Investigation, Funding acquisition, Formal analysis, Data curation. **Ahmed H. ElMesiry:** Software, Investigation, Formal analysis, Data curation. **Evans K. Quaye:** Writing – review & editing. **Zicheng Hu:** Investigation, Funding acquisition. **Ali Salem:** Investigation, Funding acquisition.

## Declaration of competing interest

The authors declare that they have no known competing financial interests or personal relationships that could have appeared to influence the work reported in this paper.

## Data Availability

The data that has been used is confidential.

## Appendix A Supplementary data

Supplementary material Image 1
