# Supplementary material for: Machine learning algorithm for estimating and optimizing the phytochemical content and physicochemical properties of okra slices in an infrared heating system
Source: Food Chem X. 2025 Jan 30;25:102248. doi: 10.1016/j.fochx.2025.102248 (PMC11838090; doi:10.1016/j.fochx.2025.102248)
Supplement: Supplementary file 1 — Supplementary material [file mmc1.docx]

| 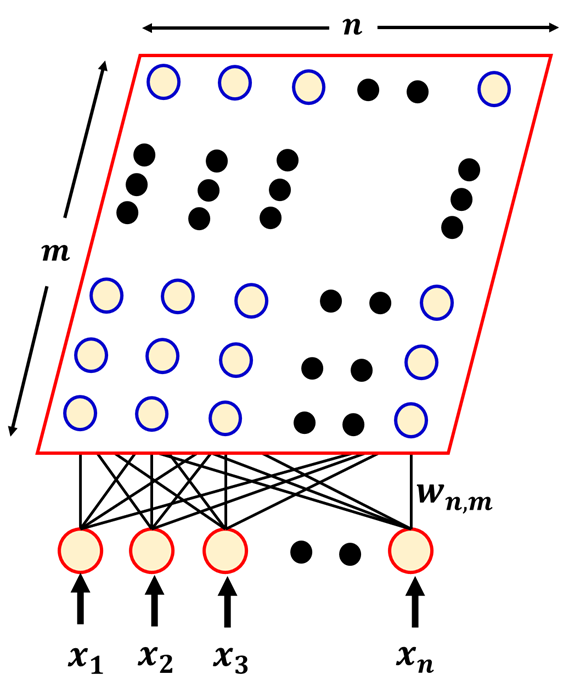 |
| --- |
| Figure S1. The construction of a Self-Organizing Map (SOM) |

Table S1. Overall performance and regression factor with different neurons and hidden layers.

|  |  | No of neurons | | | | | |
| --- | --- | --- | --- | --- | --- | --- | --- |
|  |  | 4 | 6 | 8 | 10 | 12 | 14 |
| Single hidden layer | Performance | 2.3e-2 | 2.9e-2 | 4.2e-3 | 8.2e-3 | 5.7e-3 | 7.9e-3 |
|  | Regression factor (R) | 96.89 | 97.63 | 98.01 | 98.98 | 98.27 | 98.38 |
| Two hidden layers | Performance | 1.2e-3 | 4.2e-3 | 3.3e-3 | 1.3e-4 | 8.8e-5 | 1.3e-4 |
|  | Regression factor (R) | 98.01 | 98.21 | 98.50 | 99.11 | 99.94 | 99.05 |
| Three hidden layers | Performance | 6.3e-3 | 1.1e-3 | 9.3e-4 | 6.1e-4 | 4.8e-4 | 3.4e-4 |
|  | Regression factor (R) | 97.20 | 97.92 | 97.80 | 98.01 | 98.25 | 98.02 |

Table S2. Statistical results of phytochemical and physicochemical properties of okra using ANN approach.

| Parameter | Number of hidden layer(s) | Number of layers and neurons | RMSE | R^2^ (training) | R^2^ (testing) |
| --- | --- | --- | --- | --- | --- |
| Drying time | 1 | 3-10-1 | 0.0087 | 0.9913 | 0.9936 |
|  | 1 | 3-13-1 | 0.0085 | 0.9919 | 0.9957 |
|  | 1 | 3-9-1 | 0.0084 | 0.9777 | 0.9753 |
|  | 1 | 3-7-1 | 0.0128 | 0.9934 | 0.9899 |
|  | 2 | 3-7-6-1 | 0.0495 | 0.9956 | 0.9894 |
|  | 2 | 3-17-17-1 | 0.0520 | 0.9961 | 0.9932 |
|  | 2 | 3-10-14-1 | 0.0235 | 0.9961 | 0.9850 |
|  | 2 | 3-5-5-1 | 0.0085 | 0.9968 | 0.9863 |
|  | 1 | 3-10-1 | 0.0152 | 0.9916 | 0.9831 |
|  | 1 | 3-14-1 | 0.0085 | 0.9972 | 0.9969 |
|  | 1 | 3-8-1 | 0.0463 | 0.9963 | 0.9952 |
|  | 1 | 3-5-1 | 0.0068 | 0.9981 | 0.9984 |
|  | 2 | 3-7-6-1 | 0.0056 | 0.9972 | 0.9859 |
|  | 2 | 3-7-14-1 | 0.0074 | 0.9980 | 0.9854 |
|  | 2 | 3-9-9-1 | 0.0378 | 0.9979 | 0.9952 |
|  | 2 | 3-16-16-1 | 0.2085 | 0.9959 | 0.9732 |
| Water activity | 1 | 3-10-1 | 0.0144 | 0.9956 | 0.9153 |
|  | 1 | 3-10-1 | 0.2264 | 0.9827 | 0.9416 |
|  | 1 | 3-5-1 | 0.2164 | 0.9877 | 0.9837 |
|  | 1 | 3-13-1 | 0.0070 | 0.9855 | 0.9600 |
|  | 2 | 3-6-8-1 | 0.0775 | 0.9952 | 0.9673 |
|  | 2 | 3-16-15-1 | 0.0347 | 0.9937 | 0.9888 |
|  | 2 | 3-18-18-1 | 0.0362 | 0.9953 | 0.9656 |
|  | 2 | 3-7-7-1 | 0.2084 | 0.9952 | 0.9627 |
|  | 1 | 3-10-1 | 0.0319 | 0.9932 | 0.9759 |
|  | 1 | 3-10-1 | 0.0061 | 0.9946 | 0.9858 |
|  | 1 | 3-17-1 | 0.0201 | 0.9926 | 0.9834 |
|  | 1 | 3-8-1 | 0.2410 | 0.9871 | 0.9867 |
|  | 2 | 3-10-8-1 | 0.0144 | 0.9823 | 0.9791 |
|  | 2 | 3-16-12-1 | 0.0329 | 0.9922 | 0.9781 |
|  | 2 | 3-13-11-1 | 0.0325 | 0.9926 | 0.9916 |
|  | 2 | 3-8-7-1 | 0.0170 | 0.9885 | 0.9725 |
| Vitamin C | 1 | 3-10-1 | 0.0775 | 0.9869 | 0.9481 |
|  | 1 | 3-13-1 | 0.0347 | 0.9848 | 0.9412 |
|  | 1 | 3-8-1 | 0.0362 | 0.9884 | 0.9671 |
|  | 1 | 3-11-1 | 0.2084 | 0.9847 | 0.9646 |
|  | 2 | 3-16-15-1 | 0.0319 | 0.9882 | 0.9429 |
|  | 2 | 3-14-14-1 | 0.0061 | 0.9819 | 0.9657 |
|  | 2 | 3-15-15-1 | 0.0201 | 0.9874 | 0.9721 |
|  | 2 | 3-7-7-1 | 0.2410 | 0.9831 | 0.9462 |
|  | 1 | 3-10-1 | 0.0068 | 0.9808 | 0.9702 |
|  | 1 | 3-13-1 | 0.0056 | 0.9865 | 0.9562 |
|  | 1 | 3-10-1 | 0.0074 | 0.9890 | 0.9780 |
|  | 1 | 3-20-1 | 0.0378 | 0.9684 | 0.9629 |
|  | 2 | 3-8-8-1 | 0.2085 | 0.9876 | 0.9662 |
|  | 2 | 3-15-13-1 | 0.0144 | 0.9834 | 0.9448 |
|  | 2 | 3-18-18-1 | 0.2264 | 0.9817 | 0.9484 |
|  | 2 | 3-7-6-1 | 0.2164 | 0.9859 | 0.9612 |
| TPC | 1 | 3-12-1 | 0.0070 | 0.9945 | 0.9759 |
|  | 1 | 3-9-1 | 0.0068 | 0.9935 | 0.9755 |
|  | 1 | 3-15-1 | 0.0066 | 0.9945 | 0.9672 |
|  | 1 | 3-7-1 | 0.2410 | 0.9951 | 0.9758 |
|  | 2 | 3-9-9-1 | 0.0087 | 0.9916 | 0.9619 |
|  | 2 | 3-11-10-1 | 0.0085 | 0.9901 | 0.9624 |
|  | 2 | 3-10-10-1 | 0.0084 | 0.9950 | 0.9760 |
|  | 2 | 3-8-6-1 | 0.0128 | 0.9943 | 0.9845 |
|  | 1 | 3-10-1 | 0.0495 | 0.9935 | 0.9825 |
|  | 1 | 3-13-1 | 0.0520 | 0.9934 | 0.9881 |
|  | 1 | 3-14-1 | 0.0235 | 0.9925 | 0.9704 |
|  | 1 | 3-6-1 | 0.0085 | 0.9911 | 0.9879 |
|  | 2 | 3-14-14-1 | 0.0056 | 0.9950 | 0.9773 |
|  | 2 | 3-12-11-1 | 0.0074 | 0.9952 | 0.9942 |
|  | 2 | 3-20-15-1 | 0.0378 | 0.9956 | 0.9924 |
|  | 2 | 3-9-8-1 | 0.2085 | 0.9922 | 0.9871 |
| TFC | 1 | 3-12-1 | 0.0052 | 0.9945 | 0.9759 |
|  | 1 | 3-9-1 | 0.0063 | 0.9935 | 0.9755 |
|  | 1 | 3-15-1 | 0.0061 | 0.9945 | 0.9672 |
|  | 1 | 3-7-1 | 0.0775 | 0.9951 | 0.9758 |
|  | 2 | 3-9-9-1 | 0.0235 | 0.9916 | 0.9619 |
|  | 2 | 3-11-10-1 | 0.0085 | 0.9901 | 0.9624 |
|  | 2 | 3-10-10-1 | 0.0056 | 0.9950 | 0.9760 |
|  | 2 | 3-8-6-1 | 0.0074 | 0.9943 | 0.9845 |
|  | 1 | 3-10-1 | 0.0378 | 0.9935 | 0.9825 |
|  | 1 | 3-13-1 | 0.2085 | 0.9934 | 0.9881 |
|  | 1 | 3-14-1 | 0.0052 | 0.9925 | 0.9704 |
|  | 1 | 3-6-1 | 0.0061 | 0.9911 | 0.9879 |
|  | 2 | 3-14-14-1 | 0.0201 | 0.9950 | 0.9773 |
|  | 2 | 3-12-11-1 | 0.2410 | 0.9952 | 0.9942 |
|  | 2 | 3-20-15-1 | 0.0068 | 0.9956 | 0.9924 |
|  | 2 | 3-9-8-1 | 0.0056 | 0.9922 | 0.9871 |
| Chlorophyll content | 1 | 3-10-1 | 0.0087 | 0.9913 | 0.9936 |
|  | 1 | 3-13-1 | 0.0085 | 0.9919 | 0.9957 |
|  | 1 | 3-9-1 | 0.0084 | 0.9777 | 0.9753 |
|  | 1 | 3-7-1 | 0.0128 | 0.9934 | 0.9899 |
|  | 2 | 3-7-6-1 | 0.0495 | 0.9956 | 0.9894 |
|  | 2 | 3-17-17-1 | 0.0520 | 0.9961 | 0.9932 |
|  | 2 | 3-10-14-1 | 0.0235 | 0.9961 | 0.9850 |
|  | 2 | 3-5-5-1 | 0.0085 | 0.9968 | 0.9863 |
|  | 1 | 3-10-1 | 0.0152 | 0.9916 | 0.9831 |
|  | 1 | 3-14-1 | 0.0085 | 0.9972 | 0.9969 |
|  | 1 | 3-8-1 | 0.0463 | 0.9963 | 0.9952 |
|  | 1 | 3-5-1 | 0.0068 | 0.9981 | 0.9984 |
|  | 2 | 3-7-6-1 | 0.0056 | 0.9972 | 0.9859 |
|  | 2 | 3-7-14-1 | 0.0074 | 0.9980 | 0.9854 |
|  | 2 | 3-9-9-1 | 0.0378 | 0.9979 | 0.9952 |
|  | 2 | 3-16-16-1 | 0.2085 | 0.9959 | 0.9732 |
| Rehydration ratio | 1 | 3-12-1 | 0.0070 | 0.9945 | 0.9759 |
|  | 1 | 3-9-1 | 0.0068 | 0.9935 | 0.9755 |
|  | 1 | 3-15-1 | 0.0066 | 0.9945 | 0.9672 |
|  | 1 | 3-7-1 | 0.2410 | 0.9951 | 0.9758 |
|  | 2 | 3-9-9-1 | 0.0087 | 0.9916 | 0.9619 |
|  | 2 | 3-11-10-1 | 0.0085 | 0.9901 | 0.9624 |
|  | 2 | 3-10-10-1 | 0.0084 | 0.9950 | 0.9760 |
|  | 2 | 3-8-6-1 | 0.0128 | 0.9943 | 0.9845 |
|  | 1 | 3-10-1 | 0.0495 | 0.9935 | 0.9825 |
|  | 1 | 3-13-1 | 0.0520 | 0.9934 | 0.9881 |
|  | 1 | 3-14-1 | 0.0235 | 0.9925 | 0.9704 |
|  | 1 | 3-6-1 | 0.0085 | 0.9911 | 0.9879 |
|  | 2 | 3-14-14-1 | 0.0056 | 0.9950 | 0.9773 |
|  | 2 | 3-12-11-1 | 0.0074 | 0.9952 | 0.9942 |
|  | 2 | 3-20-15-1 | 0.0378 | 0.9956 | 0.9924 |
|  | 2 | 3-9-8-1 | 0.2085 | 0.9922 | 0.9871 |

Table S3. The statistical analysis of drying time and phytochemical and physicochemical properties of okra slices by ANOVA included (P-values and F-statistics).

| \| **Drying time** \| **DF** \| **Adj SS** \| **Adj MS** \| **F-Value** \| **P-Value** \| \| --- \| --- \| --- \| --- \| --- \| --- \| \| Velocity \| 2 \| 82850 \| 41425.0 \| 4262.73 \| 0.000 \| \| Temperature \| 2 \| 107450 \| 53725.0 \| 5528.43 \| 0.000 \| \| IR \| 2 \| 107450 \| 53725.0 \| 5528.43 \| 0.000 \| \| Rep \| 2 \| 18593 \| 9296.3 \| 956.61 \| 0.000 \| \| Velocity*Temperature \| 4 \| 400 \| 100.0 \| 10.29 \| 0.000 \| \| Velocity*IR \| 4 \| 400 \| 100.0 \| 10.29 \| 0.000 \| \| Temperature*IR \| 4 \| 3250 \| 812.5 \| 83.61 \| 0.000 \| \| Velocity*Temperature*IR \| 8 \| 1400 \| 175.0 \| 18.01 \| 0.000 \| \| Error \| 52 \| 505 \| 9.7 \|  \|  \| \| Total \| 80 \| 322298 \|  \|  \|  \| |
| --- | --- | --- | --- | --- | --- | --- | --- | --- | --- | --- | --- | --- | --- | --- | --- | --- | --- | --- | --- | --- | --- | --- | --- | --- | --- | --- | --- | --- | --- | --- | --- | --- | --- | --- | --- | --- | --- | --- | --- | --- | --- | --- | --- | --- | --- | --- | --- | --- | --- | --- | --- | --- | --- | --- | --- | --- | --- | --- | --- | --- | --- | --- | --- | --- | --- | --- |
| \| **Water activity** \| **DF** \| **Adj SS** \| **Adj MS** \| **F-Value** \| **P-Value** \| \| --- \| --- \| --- \| --- \| --- \| --- \| \| Velocity \| 2 \| 80.746 \| 40.373 \| 2702.80 \| 0.000 \| \| Temperature \| 2 \| 121.541 \| 60.771 \| 4068.33 \| 0.000 \| \| IR \| 2 \| 255.946 \| 127.973 \| 8567.24 \| 0.000 \| \| Rep \| 2 \| 35.250 \| 17.625 \| 1179.91 \| 0.000 \| \| Velocity*Temperature \| 4 \| 2.117 \| 0.529 \| 35.43 \| 0.000 \| \| Velocity*IR \| 4 \| 3.993 \| 0.998 \| 66.83 \| 0.000 \| \| Temperature*IR \| 4 \| 0.791 \| 0.198 \| 13.24 \| 0.000 \| \| Velocity*Temperature*IR \| 8 \| 0.916 \| 0.114 \| 7.66 \| 0.000 \| \| Error \| 52 \| 0.777 \| 0.015 \|  \|  \| \| Total \| 80 \| 502.077 \|  \|  \|  \| |
| \| **Rehydration ratio** \| **DF** \| **Adj SS** \| **Adj MS** \| **F-Value** \| **P-Value** \| \| --- \| --- \| --- \| --- \| --- \| --- \| \| Velocity \| 2 \| 0.56696 \| 0.283478 \| 5076.95 \| 0.000 \| \| Temperature \| 2 \| 0.41362 \| 0.206811 \| 3703.89 \| 0.000 \| \| IR \| 2 \| 0.68116 \| 0.340578 \| 6099.59 \| 0.000 \| \| Rep \| 2 \| 0.41800 \| 0.209000 \| 3743.09 \| 0.000 \| \| Velocity*Temperature \| 4 \| 0.03171 \| 0.007928 \| 141.98 \| 0.000 \| \| Velocity*IR \| 4 \| 0.03298 \| 0.008244 \| 147.65 \| 0.000 \| \| Temperature*IR \| 4 \| 0.00431 \| 0.001078 \| 19.30 \| 0.000 \| \| Velocity*Temperature*IR \| 8 \| 0.01136 \| 0.001419 \| 25.42 \| 0.000 \| \| Error \| 52 \| 0.00290 \| 0.000056 \|  \|  \| \| Total \| 80 \| 2.16299 \|  \|  \|  \| |
| \| **Shrinkage ratio** \| **DF** \| **Adj SS** \| **Adj MS** \| **F-Value** \| **P-Value** \| \| --- \| --- \| --- \| --- \| --- \| --- \| \| IR (W/m²) \| 2 \| 0.031267 \| 0.015633 \| 195.64 \| 0.000 \| \| V (m/s) \| 2 \| 0.009600 \| 0.004800 \| 60.07 \| 0.000 \| \| T (°C) \| 2 \| 0.023267 \| 0.011633 \| 145.58 \| 0.000 \| \| Error \| 74 \| 0.005913 \| 0.000080 \|  \|  \| \| Lack-of-Fit \| 20 \| 0.001267 \| 0.000063 \| 0.74 \| 0.772 \| \| Pure Error \| 54 \| 0.004647 \| 0.000086 \|  \|  \| \| Total \| 80 \| 0.070046 \|  \|  \|  \| |
| \| **Hardness** \| **DF** \| **Adj SS** \| **Adj MS** \| **F-Value** \| **P-Value** \| \| --- \| --- \| --- \| --- \| --- \| --- \| \| IR (W/m²) \| 2 \| 162.717 \| 81.358 \| 191.00 \| 0.000 \| \| V (m/s) \| 2 \| 184.464 \| 92.232 \| 216.53 \| 0.000 \| \| T (°C) \| 2 \| 226.347 \| 113.174 \| 265.69 \| 0.000 \| \| Error \| 74 \| 31.521 \| 0.426 \|  \|  \| \| Lack-of-Fit \| 20 \| 3.978 \| 0.199 \| 0.39 \| 0.988 \| \| Pure Error \| 54 \| 27.543 \| 0.510 \|  \|  \| \| Total \| 80 \| 605.049 \|  \|  \|  \| |
| \| **Color** \| **DF** \| **Adj SS** \| **Adj MS** \| **F-Value** \| **P-Value** \| \| --- \| --- \| --- \| --- \| --- \| --- \| \| IR (W/m²) \| 2 \| 171.743 \| 85.872 \| 278.13 \| 0.000 \| \| V (m/s) \| 2 \| 236.269 \| 118.135 \| 382.62 \| 0.000 \| \| T (°C) \| 2 \| 237.460 \| 118.730 \| 384.55 \| 0.000 \| \| Error \| 74 \| 22.847 \| 0.309 \|  \|  \| \| Lack-of-Fit \| 20 \| 2.032 \| 0.102 \| 0.26 \| 0.999 \| \| Pure Error \| 54 \| 20.815 \| 0.385 \|  \|  \| \| Total \| 80 \| 668.320 \|  \|  \|  \| |
| \| **Browning index** \| **DF** \| **Adj SS** \| **Adj MS** \| **F-Value** \| **P-Value** \| \| --- \| --- \| --- \| --- \| --- \| --- \| \| IR (W/m²) \| 2 \| 171.743 \| 85.872 \| 348.61 \| 0.000 \| \| V (m/s) \| 2 \| 157.535 \| 78.767 \| 319.77 \| 0.000 \| \| T (°C) \| 2 \| 237.460 \| 118.730 \| 482.01 \| 0.000 \| \| Error \| 74 \| 18.228 \| 0.246 \|  \|  \| \| Lack-of-Fit \| 20 \| 2.032 \| 0.102 \| 0.34 \| 0.995 \| \| Pure Error \| 54 \| 16.196 \| 0.300 \|  \|  \| \| Total \| 80 \| 584.966 \|  \|  \|  \| |
| \| **Vitamin C** \| **DF** \| **Adj SS** \| **Adj MS** \| **F-Value** \| **P-Value** \| \| --- \| --- \| --- \| --- \| --- \| --- \| \| IR (W/m²) \| 2 \| 150.544 \| 75.272 \| 94.69 \| 0.000 \| \| V (m/s) \| 2 \| 29.788 \| 14.894 \| 18.74 \| 0.000 \| \| T (°C) \| 2 \| 227.311 \| 113.656 \| 142.98 \| 0.000 \| \| Error \| 74 \| 58.824 \| 0.795 \|  \|  \| \| Lack-of-Fit \| 20 \| 4.887 \| 0.244 \| 0.24 \| 0.999 \| \| Pure Error \| 54 \| 53.936 \| 0.999 \|  \|  \| \| Total \| 80 \| 466.467 \|  \|  \|  \| |
| \| **TFC** \| **DF** \| **Adj SS** \| **Adj MS** \| **F-Value** \| **P-Value** \| \| --- \| --- \| --- \| --- \| --- \| --- \| \| IR (W/m²) \| 2 \| 29.496 \| 14.7482 \| 456.62 \| 0.000 \| \| V (m/s) \| 2 \| 16.697 \| 8.3485 \| 258.47 \| 0.000 \| \| T (°C) \| 2 \| 21.403 \| 10.7013 \| 331.32 \| 0.000 \| \| Error \| 74 \| 2.390 \| 0.0323 \|  \|  \| \| Lack-of-Fit \| 20 \| 1.264 \| 0.0632 \| 3.03 \| 0.001 \| \| Pure Error \| 54 \| 1.126 \| 0.0209 \|  \|  \| \| Total \| 80 \| 69.986 \|  \|  \|  \| |
| \| **TFC** \| **DF** \| **Adj SS** \| **Adj MS** \| **F-Value** \| **P-Value** \| \| --- \| --- \| --- \| --- \| --- \| --- \| \| IR (W/m²) \| 2 \| 29.496 \| 14.7482 \| 456.62 \| 0.000 \| \| V (m/s) \| 2 \| 16.697 \| 8.3485 \| 258.47 \| 0.000 \| \| T (°C) \| 2 \| 21.403 \| 10.7013 \| 331.32 \| 0.000 \| \| Error \| 74 \| 2.390 \| 0.0323 \|  \|  \| \| Lack-of-Fit \| 20 \| 1.264 \| 0.0632 \| 3.03 \| 0.001 \| \| Pure Error \| 54 \| 1.126 \| 0.0209 \|  \|  \| \| Total \| 80 \| 69.986 \|  \|  \|  \| |
